# Orthogonal CRISPR systems for targeted integration and multiplex base editing enable nonviral engineering of allogeneic CAR-T cells

**DOI:** 10.1016/j.ymthe.2025.08.032

**Published:** 2025-08-26

**Authors:** Nanna S. Mikkelsen, Sujan Ravendran, Amalie D. Broksø, Sigrid Fu Skjelbostad, Maya G. Pedersen, Hongyu Fang, Thorkild Terkelsen, Martin Kristian Thomsen, Rasmus O. Bak

**Affiliations:** 1 Department of Biomedicine, Aarhus University, 8000 Aarhus C, Denmark; 2 Department of Clinical Medicine, Aarhus University, 8000 Aarhus C, Denmark; 3 Department for Clinical Genetics, Aarhus University Hospital, 8200 Aarhus C, Denmark

**Keywords:** CRISPR-Cas, orthogonal CRISPR systems, CAR, chimeric antigen receptor, base editing, SaCas9, Cas9 orthologs, ssDNA, CTS, regnase-1

## Abstract

Multiple genomic modifications, including targeted transgene integrations and knockouts, may be required to develop potent, allogeneic chimeric antigen receptor (CAR)-T cell therapies. Conventional CRISPR-Cas systems generate double-strand breaks (DSBs) associated with genomic rearrangements and genotoxicities. DSB-free base editing reduces these risks. Here, we facilitate multiplex editing by combining *Staphylococcus aureus* Cas9 (SaCas9) mRNA base editors for DSB-free knockout of *B2M* and *REGNASE-1* with *Streptococcus pyogenes* Cas9 nucleases for targeted integration of an anti-CD19 CAR transgene at the T cell receptor α constant locus. Combined, these edits have been reported to generate safer allogeneic CAR-T cells with enhanced activity and persistence. We demonstrate multiplex gene editing in primary human T cells with *B2M* and *REGNASE-1* base editing frequencies reaching 66% and 84%, respectively, while integrating the anti-CD19 CAR transgene in up to 36% or 71% of cells using nonviral single-stranded DNA repair templates or viral vector templates (AAV6), respectively. Importantly, no detrimental effects on CAR-T cell function were observed *in vitro* or *in vivo*, and knockout by base editing reduced rates of balanced chromosomal translocations by 210-fold. This orthogonal CRISPR-Cas engineering approach represents a novel and safer strategy for nonviral, multiplexed genetic engineering of CAR-T cells.

## Graphical abstract

**Figure:**
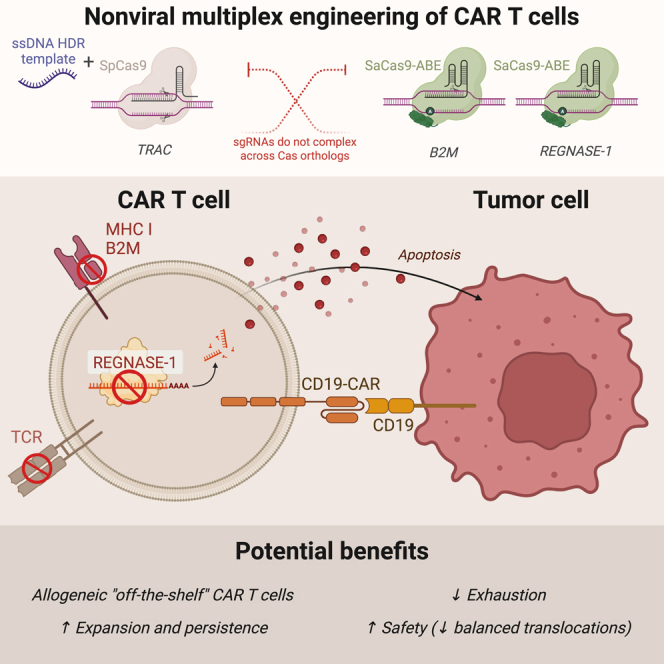

## Teaser

Bak and colleagues generate multiplex edited CAR-T cells by combining DNA double-stranded break-free base editing knockout of *B2M* and *REGNASE-1* using a *Staphylococcus aureus* Cas9 base editor with nonviral targeted integration of an anti-CD19 CAR transgene into the T cell receptor α constant locus using a *Streptococcus pyogenes* nuclease, which demonstrates efficient multiplex editing and reduced translocations.

## Introduction

Chimeric antigen receptor (CAR)-T cell therapies, in which T cells are genetically engineered to recognize specific tumor surface antigens and independently mediate a T cell response, have led to tremendous progress in the treatment of relapsed or refractory B cell malignancies.^1^^,^^2^^,^^3^^,^^4^ This has resulted in regulatory approval of multiple cellular products and encouraged the development of future therapies against a variety of cancer types and other chronic diseases.^5^^,^^6^^,^^7^^,^^8^ However, widespread application of CAR-T cell therapies are limited by the autologous nature of the T cells, the risk of poor cell quantity and quality due to extensive prior chemotherapy, substantial manufacturing time and cost, and an immunosuppressive tumor microenvironment impeding CAR-T cell activity.^9^ Genetically engineered universal “off-the-shelf” allogeneic CAR-T cells derived from healthy donors could resolve these limitations and provide a considerable advantage compared to autologous T cells.^10^ Previously, allogeneic CAR-T cells have been used in clinical trials,^11^^,^^12^ but require strategies to prevent graft-versus-host disease (GvHD) and CAR-T cell rejection by recipient immune cells. Elimination of the αβ T cell receptor (TCR) through T cell receptor α constant (*TRAC*) gene disruption prevents reactivity of the donor T cells against the recipient and thereby abrogate GvHD.^9^ Elimination of the *B2M* gene would abolish major histocompatibility complex (MHC) class I (histocompatibility leukocyte antigen-I [HLA-I]) expression, thereby minimizing immunogenicity and rejection by the host immune system.^13^ Targeted nucleases like the CRISPR-Cas system are being utilized for such multiplex gene editing.^14^ The CRISPR-Cas system introduces DNA double-stranded breaks (DSBs) at precise genomic locations that can be resolved by endogenous DSB repair processes. This can be exploited for genome engineering either by induction of insertions or deletions (indels) by error-prone non-homologous end joining or for precise targeted integration of a transgene by homology-directed repair (HDR) using a repair template carrying homologous sequences from the regions flanking the DSB.^15^

In the pursuit of engineering allogeneic CAR-T cells, several studies have combined multiplex genome editing to knock out relevant genes (e.g., *TRAC* and *B2M* genes, as well as genes to increase persistence and resist chemotherapeutic agents and inhibitory tumor signals) in combination with retroviral or lentiviral transduction for genomic integration of the CAR transgene.^16^^,^^17^^,^^18^^,^^19^^,^^20^ However, introducing multiple DSBs simultaneously can cause various genomic rearrangements, genomic instability triggering programmed cell death, and chromosomal translocations.^21^^,^^22^^,^^23^^,^^24^^,^^25^^,^^26^ Such consequences have been observed for multiplex edited T cells, for example, in TCR-engineered T cells with multiplex editing at three loci (*TRAC*, *TRBC*, and *PDCD1*), which showed translocations and genomic rearrangements both pre- and post-infusion in all patients.^27^

Newer CRISPR-Cas genome-editing tools, like base editing and prime editing, do not rely on DSBs and therefore provide safer means to modify genes.^28^^,^^29^^,^^30^^,^^31^ Base editing has demonstrated highly efficient single base pair editing at specific genomic loci by combining the programmability of the CRISPR-Cas system with DNA deaminases. For example, adenine base editors (ABEs) utilize a Cas9 nickase coupled to a DNA deaminase to introduce A-T to G-C point mutations.^29^ Base editing can be utilized to disrupt gene expression by introducing point mutations to disrupt a start codon,^32^ introduce premature stop codons,^33^ or disrupt splice sites, potentially resulting in exon skipping or intron retention and nonsense-mediated decay (NMD).^34^ Thereby, multiplex base editing can be achieved with fewer undesired editing outcomes and with minimal safety concerns otherwise associated with standard nucleases. Several studies have demonstrated multiplex base editing knockout combined with lentiviral gene transfer to engineer enhanced CAR-T cells.^35^^,^^36^^,^^37^ This approach has even been proven to be efficient in one clinical trial.^38^

Nonviral approaches are attractive for future clinical applications of CAR-T cells since viral gene therapies have been associated with insertional mutagenesis in the case of retroviral and lentiviral vectors, immunogenicity, p53 activation, and other toxicities.^39^^,^^40^^,^^41^ Furthermore, viral approaches suffer from high manufacturing complexity and extensive cost.^40^ CRISPR-Cas-mediated site-specific integration of a CAR gene into the *TRAC* locus has been shown to facilitate both TCR ablation and physiological and uniform CAR expression from the endogenous promoter.^42^^,^^43^ In addition, site-specific integration reduces the risk of retroviral insertional mutagenesis associated with integrating viral vectors. Vectors based on adeno-associated virus serotype 6 (AAV6) have constituted the preferred HDR template in T cells, but to circumvent the elaborate viral vector manufacturing, both linear and circular double-stranded DNA (dsDNA) and single-stranded DNA (ssDNA) have been used.^44^^,^^45^ However, these approaches are less efficient when integrating larger sequences required for CAR-T cell therapy often exceeding 1,500 bp.

To minimize toxicity related to dsDNA and to increase HDR efficiency, ssDNA HDR templates have been utilized as these have demonstrated to be less toxic compared to dsDNA.^44^^,^^46^^,^^47^ While shorter ssDNA HDR templates, like short single-stranded oligodeoxynucleotides, have previously achieved high integration efficiencies,^44^ longer ssDNAs are less efficient. However, Shy et al. developed a hybrid HDR template consisting of long ssDNAs flanked by short regions of dsDNA containing Cas9 target sequences (CTSs), resulting in reduced toxicity and increased integration efficiency. The CTS consists of a truncated Cas9-binding sequence that allows Cas9 binding but not cutting, which facilitates increased nuclear import of the HDR template by the nuclear localization sequences on Cas9, thereby enhancing integration efficiencies.^47^^,^^48^^,^^49^

Here, we demonstrate engineering of multiplex edited CAR-T cells in a single step using adenine base editing for targeted knockout of relevant genes and Cas9 nuclease-mediated targeted integration of an anti-CD19 CAR transgene into the *TRAC* locus by HDR using repair templates delivered by AAV6 vectors or as nonviral ssDNA with CTSs. To seamlessly combine base editing knockout and HDR-based integration in T cells in a single engineering step, we apply two different Cas9 orthologs, *Staphylococcus aureus* Cas9 (SaCas9) and *Streptococcus pyogenes* Cas9 (SpCas9) that operate independently without exchange of the single-guide RNA (sgRNA). To the best of our knowledge, SaCas9-mediated base editing in primary cells like primary human T cells, delivered as an all-RNA system, as well as the combination of multiplex base editing with targeted integration using long ssDNA as an HDR template for integration, have not been done previously. We demonstrate efficient, multiplexed CAR-T cell engineering using these platforms with drastically reduced formation of translocations when applying base editing for knockout. We believe that this novel platform constitutes a rapid and efficient multiplex genome-editing approach for engineering allogeneic CAR-T cells with engineered desirable features.

## Results

### SaCas9-mediated base editing

To avoid exchange between the sgRNAs used for HDR-based transgene integration and the sgRNAs used for knockout by base editing, we chose to allocate the canonical SpCas9 for HDR, while using an SaCas9 nickase for base editing. We have previously demonstrated efficient use of these two orthogonal CRISPR systems for simultaneous gene activation and repression without observing sgRNA exchange.^50^^,^^51^ SaCas9 recognizes a 5′-NNGRRT-3′ protospacer-adjacent motif (PAM) and has an optimal spacer length of 21 nt.^52^ It has previously demonstrated high gene-editing efficiencies^52^^,^^53^ also when delivered as mRNA in an all-RNA system.^54^ Various improved BEs have been developed for SaCas9-mediated base editing and have shown efficient editing in cancer cell lines following plasmid transfection. However, SaCas9 mRNA BEs have so far not been developed, nor has SaCas9 base editing been demonstrated in primary human cells. We therefore chose the most efficient plasmid-based ABE, SaKKH-ABE8e, which recognizes a more flexible 5′-NNNRRT-3′ PAM,^55^ for generating the mRNA BE.

**Figure 1:**
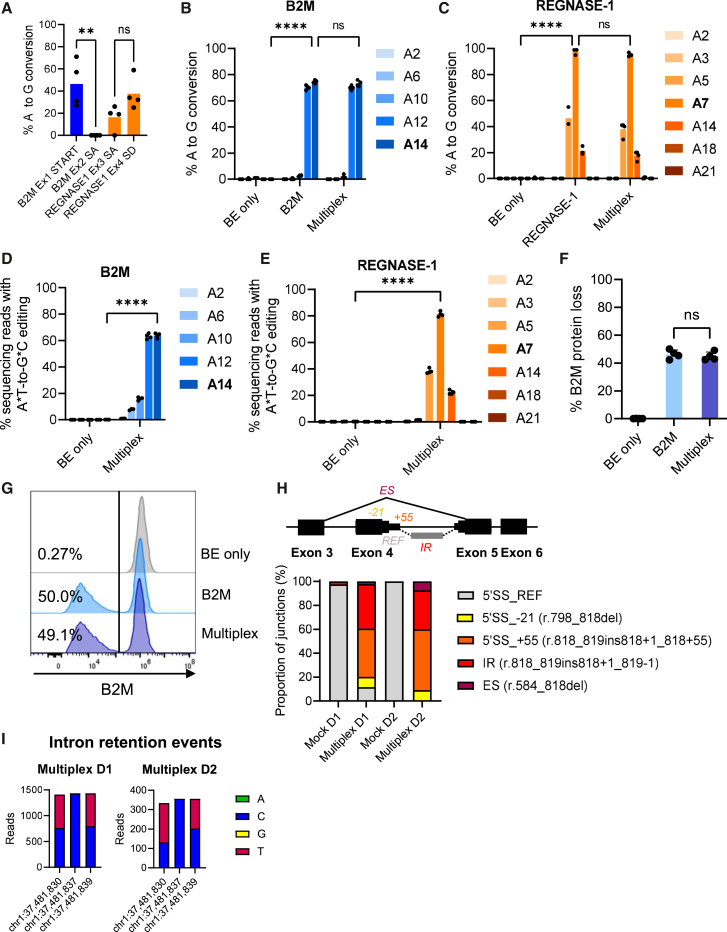
SaCas9-mediated base editing (A) Test of designed SaCas9 adenine base editor (ABE) sgRNAs. K562 cells were transfected with plasmids encoding the BE and an sgRNA, genomic DNA was Sanger sequenced, and base editing was assessed by the Base Editing Analysis Tool (BEAT). Ex, exon; SA, splice acceptor disrupting; SD, splice donor disrupting; START, start codon disrupting. (B–E) Sanger sequencing BEAT analysis (B and C) or NGS CRISPresso2 analysis (D and E) of *B2M* and *REGNASE-1* editing in T cells. Target adenines are highlighted in bold, and surrounding adenines are included in the analysis. (F) B2M protein loss assessed by flow cytometry in cells receiving only the BE (BE only), the BE and the *B2M* sgRNA (B2M), or the BE and both *B2M* and *REGNASE-1* sgRNAs (Multiplex). (G) Representative flow cytometry histograms demonstrating B2M protein loss. (H) Top: schematic illustration of splice outcomes following base editing, with alternative splice donor sites and three different splice acceptor sites in intron 4 collapsed for simplicity. Bottom: distribution of RNA-seq reads in two different T cell donors (D1 and D2) supporting use of the reference splice donor site in mock samples (5′SS_REF), and alternative splicing following base editing with the use of a splice donor site 21 bp upstream in exon 4 (5′SS_−21), an alternative donor site 55 bp downstream in intron 4 (5′SS_+55), complete intron 4 retention (IR), or exon 4 skipping (ES). (I) For all intron inclusion events (partial or complete intron retention defined as reads mapping at the end of exon 4 and at the beginning of intron 4), allele frequencies are shown as read counts for multiplex base-edited cells from two individual T cell donors. Genomic positions correspond to the following positions in the sgRNA spacer sequence: chr1:37,481,830 = A14; chr1:37,481,837 = A7 (on-target); chr1:37,481,839 = A5 (counted from the PAM distal end). Note that target adenines are on the antisense (template) DNA strand, and successful base editing (A-to-G) will result in T-to-C changes in the corresponding cDNA. Bars represent means. Statistical analysis of base editing data was performed using one-way ANOVA. Significance based on *p* values was determined as follows: ns, not significant; ∗*p* < 0.05; ∗∗*p* < 0.01; ∗∗∗*p* < 0.001; ∗∗∗∗*p* < 0.0001. *N* = 4 individual K562 cell experiments or individual T cell donors.

We initially employed plasmid-based transfection to screen a panel of sgRNAs for knockout of genes previously implicated in the generation of allogeneic or functionally enhanced CAR-T cells. For proof of concept, we focused on two genes: *B2M*, to eliminate MHC class I, and *REGNASE-1* (official gene name *ZC3H12A*), reported to enhance CAR-T cell persistence and activity by mitigating exhaustion, particular in patients with cancer where T cells are exposed to constant antigen expression and/or various inflammatory signals.^56^^,^^57^^,^^58^^,^^59^ Two sgRNAs were designed for each knockout (sgRNA sequences are listed in Table S1). sgRNAs potentially resulting in a knockout were designed manually using the CRISPR RGEN BE-Designer web tool.^60^ For each target locus, sgRNAs were chosen based on their ability to target all or most isoforms, to target early exons, and only requiring editing of one base to achieve the desired knockout by targeting the start codon or splice sites. K562 cells were transfected with plasmids encoding individual sgRNAs and SaKKH-ABE8e followed by Sanger sequencing of each target locus 2–3 days post-transfection to analyze base editing efficiencies using the Base Editing Analysis Tool (BEAT).^61^ Highest on-target base editing efficiencies were obtained at an average of 47% for the *B2M* locus by disrupting the start codon and at an average of 38% for the *REGNASE-1* locus by disrupting a splice donor site (Figure 1A). These sgRNAs with a 21-nt spacer sequence were chosen for all subsequent experiments.

### Exploring RNA-based delivery for SaCas9 multiplex base editing in T cells

To generate *in vitro* transcribed (IVT) SaKKH-ABE8e mRNA, we cloned an *in vitro* transcription plasmid carrying the promoter sequence for the T7 RNA polymerase, followed by the SaKKH-ABE8e open reading frame and a 50-bp poly(A), immediately followed by a unique restriction site to enable run-off transcription. mRNA was produced by IVT, verified on a bioanalyzer (Figure S1), and electroporated with synthetically modified sgRNAs targeting *B2M* and/or *REGNASE-1* into primary human T cells. Base editing frequencies measured by Sanger sequencing showed on-target A-to-G editing at high efficiencies with an average base conversion efficiency of 75% for *B2M* (Figure 1B). The most dominant bystander edit was at position 12 (A12) causing a missense mutation (p.Ser2Pro). The other potential bystander editing positions showed little to no editing and all introduce either silent or missense mutations (A2: p.Val5Ala, A6: p.Ser4Pro, and A10: p.Ser2Ser). For *REGNASE-1*, A-to-G base editing was achieved at even higher efficiency, reaching an average of 98% (Figure 1C). Bystander editing was observed mainly on A5, which is not expected to have implications as A5 is localized in the intron outside the canonical splice site motif. Lower bystander editing frequencies were observed on A14 (p.Asn271Asn), A18 (p.Val270Ala), and A21 (p.Phe269Ser). Since the aim was knockout to abrogate target gene expression, bystander edits causing missense mutations are of low potential risk and would need to occur without target base editing and confer a gain of function to be of concern. Simultaneous multiplex base editing of both *B2M* and *REGNASE-1* by delivering both sgRNAs yielded editing frequencies of both genes that were comparable to those observed for individual sgRNA delivery (Figures 1B and 1C). Amplicon-based next-generation sequencing (NGS) of the same samples confirmed efficient base editing but displayed slightly lower on-target base-editing rates reaching 64% for *B2M* and 81% for *REGNASE-1* (Figures 1D and 1E). Bystander non-canonical A-to-T and A-to-C conversions were minimal (<0.7% and 6% in a single sample), and no indel formation was observed at either target locus (Figures S2A–S2D).

Assessment of functional protein loss following base-editing knockout showed B2M protein loss in an average of 46% of cells, as measured by flow cytometry (Figures 1F and 1G). Attempts to evaluate REGNASE-1 loss by either quantitative reverse-transcription PCR (RT-qPCR) or western blotting were unsuccessful due to isoform differences at the target site and unspecific binding of multiple tested antibodies, respectively. However, bulk RNA sequencing (RNA-seq) of multiplex base-edited cells, following cycloheximide treatment to inhibit NMD, confirmed successful disruption of the splice donor site, resulting in abnormal REGNASE-1 mRNA splicing. In two T cell donors, only 5.75% of transcripts retained normal splicing (11.5% and 0%, respectively) (Figures 1H, S3A, and S3B). None of the aberrant splice forms were predicted to encode functional REGNASE-1 protein. The two predominant splicing events were (1) partial retention of a 55-bp segment of intron 4 due to the use of an alternative splice donor site and (2) complete retention of intron 4. Both events result in the same premature stop codon, p.(Phe274Tyrfs∗8). All intron retention reads harbored the intended base edit at the on-target position (A7; chr1:37,481,837), confirming that splicing disruption was a direct consequence of base editing (Figures 1I and S3C).

These results demonstrate that the SaCas9 ABE, SaKKH-ABE8e, is functional in an all-RNA format and enables high frequencies of multiplex base editing of both *B2M* and *REGNASE-1* in primary human T cells.

### Engineering CAR-T cells by utilizing recombinant AAV6 for HDR template delivery for targeted gene integration in T cells

**Figure 2:**
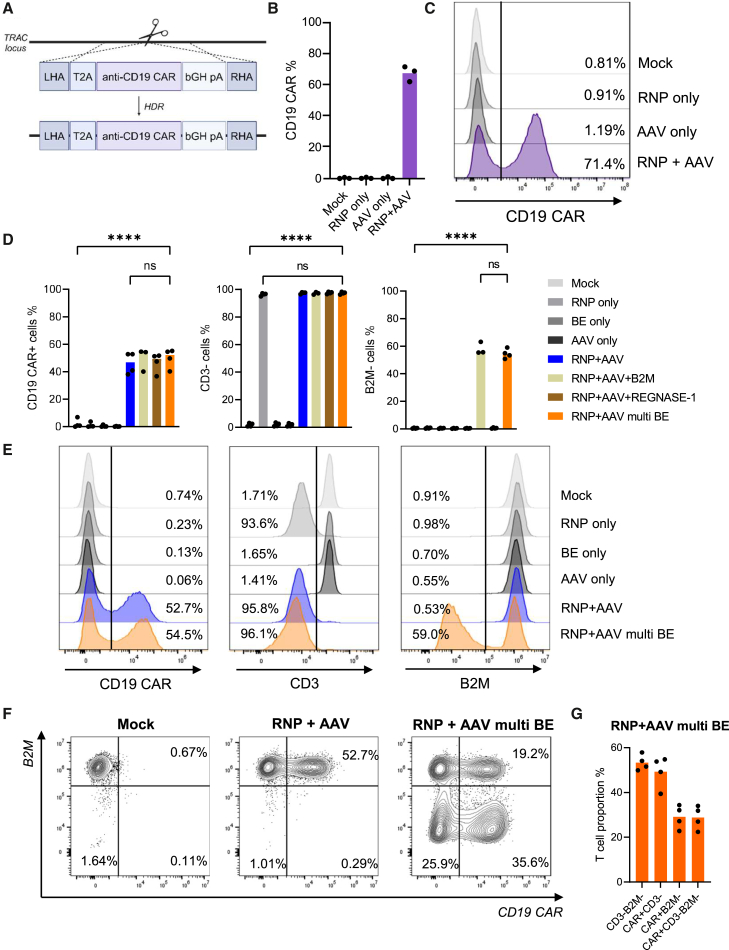
CD19-CAR transgene integration by AAV6 delivery of HDR templates and engineering of multiplex edited CAR-T cells (A) Schematic of integration of HDR template. LHA, left homology arm; pA, poly(A); RHA, right homology arm. (B) Frequencies assessed by flow cytometry of T cells expressing the anti-CD19 CAR transgene 4 days following RNP electroporation and AAV6 transduction of primary human T cells. (C) Representative flow cytometry histograms showing CAR expression. (D) Flow cytometry data showing CAR expression, CD3 knockout, and *B2M* knockout in T cells. One data point for the RNP+AAV+B2M condition was lost. (E) Representative flow cytometry histograms showing editing outcomes for CAR integration, CD3 knockout, and *B2M* knockout. (F) Representative flow cytometry plots showing multiplex editing with simultaneous CAR integration and *B2M* gene knockout. (G) Summary of cells with double and triple editing frequencies based on flow cytometry data. Bars represent means. Statistical analyses were performed using one-way ANOVA. Significance based on *p* values was determined as follows: ns, not significant; ∗*p* < 0.05; ∗∗*p* < 0.01; ∗∗∗*p* < 0.001; ∗∗∗∗*p* < 0.0001. *N* = 3–4 individual T cell donors.

Following the establishment of RNA-based delivery of SaCas9 BEs, which demonstrate high editing efficiencies, we next established HDR-based integration of the anti-CD19 CAR into exon 1 of the *TRAC* locus for simultaneous TCR knockout, as previously reported.^42^ Human primary T cells were electroporated with recombinant SpCas9 protein precomplexed with a previously reported *TRAC* sgRNA to form a ribonucleoprotein (RNP) complex.^43^^,^^62^ Electroporation was directly followed by AAV6 transduction delivering the HDR template to integrate the anti-CD19 CAR transgene. The CD19 CAR transgene is a promoterless construct relying on seamless integration and expression from the endogenous TCR α-chain promoter (Figure 2A). Integration was assessed by flow cytometry by measuring anti-CD19 CAR expression in the engineered cells, demonstrating an average of 67% CAR^+^ T cells (Figures 2B and 2C).

### Multiplex engineering of allogeneic CAR-T cells by simultaneous base editing and CAR transgene integration into the *TRAC* locus

Following the establishment of SaCas9 base editing and targeted integration using AAV6 repair templates, we next aimed to combine these systems to engineer multiplexed, engineered, allogeneic CAR-T cells. Human primary T cells were electroporated with SaKKH-ABE8e mRNA, synthetically modified sgRNAs targeting *B2M* and *REGNASE-1* for knockout, and a SpCas9 RNP complex targeting the *TRAC* locus, followed by transduction with recombinant AAV6 (rAAV6) HDR templates to integrate the promoter-less anti-CD19 CAR transgene into the *TRAC* locus. In these experiments, we increased the amount of the BE and sgRNAs to 3 μg each, as this was observed to increase on-target base editing (Figures S4A–S4C). *B2M* knockout assessed by flow cytometry demonstrated knockout efficiencies comparable to those obtained from cells with a single base editing knockout, resulting in an average of 54% cells with loss of B2M (Figures 2D and 2E). Multiplex base editing did not interfere with CD19 CAR integration and expression when compared to a control omitting the base editing components, achieving an average of 52% of T cells expressing the CAR (Figures 2D and 2E). Targeted integration and indel formation at the *TRAC* locus resulted in an average of 97% TCR^−^ cells, thus demonstrating the high efficiency of the *TRAC* sgRNA, which is crucial to avoid GvHD for treatment with allogeneic CAR-T cell products. The total efficiency of TCR knockout was also not negatively impacted when including the two BEs, nor was the knockout efficiency of *B2M* affected in the multiplex setting. Overall, this yielded up to 36% T cells being CAR^+^B2M^−^ and just as many being CAR^+^B2M^−^TCR^−^ (Figures 2F and 2G). Importantly, neither cell viability nor expansion potential of the T cells was significantly affected by the multiplex editing procedure (Figures S5A–S5C).

In conclusion, we demonstrate the successful integration of an anti-CD19 CAR into human T cells using AAV HDR templates with simultaneous base editing to knock out two genes.

### Multiplex base editing of CAR-T cells does not impair CAR-T cell function *in vitro* or *in vivo*

**Figure 3:**
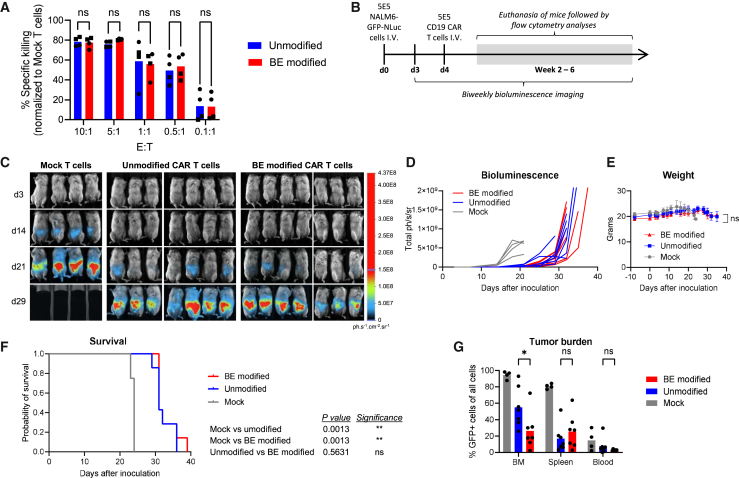
Assessment of cytotoxicity and antitumor activity *in vitro* and *in vivo* of multiplex edited CD19 CAR-T cells (A) Cytotoxicity of unmodified and BE-modified CD19 CAR-T cells was assessed by a co-culture killing assay. CD19^+^ NALM6 target cells expressing GFP were co-cultured with effector CAR-T cells at various effector-to-target ratios for 24 h. (B) Schematic of the experimental procedure for *in vivo* studies. The xenograft model was established by intravenous injection of 5E5 NALM6-GFP-NLuc cells. Mice were randomly assigned to treatment groups on day 4, with four receiving no treatment (mock) and seven in each group receiving either unmodified or BE-modified CD19 CAR-T cells. All mice were monitored by bioluminescence imaging biweekly and euthanized at humane endpoints or at a defined bioluminescence signal. (C) Bioluminescence images from the indicated days post-NALM6 injection (Figure S7 shows the full image set). (D) Quantified bioluminescence signals from individual mice, with a display cutoff set at <2E−9 ph/s/sr. (E) Mouse body weight throughout the study. (F) Kaplan-Meier survival curves for the different treatment groups. Log rank *p* values (Mantel-Cox test) was used to determine significance between groups. (G) Terminal flow cytometry analysis of bone marrow (BM), spleen, and peripheral blood (Blood) assessing the frequency of GFP^+^ NALM6 cells corresponding to the disease burden in each tissue. Bars represent means. Statistical analyses of *in vitro* cytotoxicity assays and *in vivo* tumor burden were performed using two-way ANOVA. Statistical analyses of weight comparing unmodified and BE-modified groups were performed using unpaired t tests. Significance based on *p* values was determined as follows: ns, not significant; ∗*p* < 0.05; ∗∗*p* < 0.01; ∗∗∗*p* < 0.001; ∗∗∗∗*p* < 0.0001. *N* = 2 biological replicates from two individual T cell donors (for the *in vitro* experiment, data points from each donor are designated by circles and squares, respectively) (one donor marked with a circle and one donor marked with a square). *N* = 4–7 individual mice for the *in vivo* experiments.

To assess the impact of multiplex base editing on CD19 CAR-T cells in terms of phenotype and cytotoxic potential, we compared CD19 CAR-T cells (unmodified) with multiplex base-edited CD19 CAR-T cells (BE modified). Efficient editing was confirmed by *B2M* and *REGNASE-1* sequencing and CD19 CAR, CD3, and B2M phenotyping by flow cytometry (Figures S6A and S6B). Cytotoxicity of unmodified and BE-modified CD19 CAR-T cells was assessed by *in vitro* co-culture killing assays with CD19-expressing NALM6 cells. Both unmodified and BE-modified CD19 CAR-T cells efficiently killed NALM6 cells with no significant differences across different effector-to-target (E:T) ratios (Figure 3A). Interferon-γ (IFN-γ) and tumor necrosis factor α (TNF-α) secretion were quantified by enzyme-linked immunosorbent assay (ELISA) at the 5:1 E:T ratio, revealing no differences between unmodified and BE-modified CD19 CAR-T cells (Figure S6C). Similarly, phenotypic analyses showed no differences in differentiation state (CCR7 and CD45RA), activation status (CD69, CD25, and HLA-DR), or exhaustion markers (LAG3 and PD1) (Figure S6D).

To evaluate the *in vivo* cytotoxic potential of unmodified and BE-modified CD19 CAR-T cells, we assessed anti-leukemic activity in a xenograft model using immunodeficient mice. Mice were intravenously injected with NALM6 cells expressing GFP and NanoLuc luciferase (NLuc), followed 4 days later by the administration of mock-electroporated T cells, unmodified CD19 CAR-T cells, or BE-modified CD19 CAR-T cells (Figure 3B). Both unmodified and BE-modified CD19 CAR-T cells delayed leukemia progression as assessed by *in vivo* bioluminescence imaging and mouse body weight (Figures 3C–3E and S7), resulting in significantly prolonged survival of mice treated with CAR-T cells (Figure 3F). No differences between unmodified or BE-modified CD19 CAR-T cells were observed. At terminal analyses of mice, disease load was assessed by measuring the frequencies of GFP^+^ cells in the bone marrow, spleen, and peripheral blood (PB) by flow cytometry. We observed a reduced leukemic load in the bone marrow of mice treated with BE-modified CAR-T cells compared to mice treated with unmodified CAR-T cells, but no differences in the spleen or blood were observed (Figure 3G). Also, no differences were found between the two groups in the proportions of CAR^+^, CD4^+^, or CD8^+^ T cells (Figure S6E). Finally, we analyzed potential antigen escape by measuring the frequency of CD19^−^ cells among all GFP^+^ cells but did not detect any significant differences between groups (Figure S6F).

Combined, these *in vitro* and *in vivo* experiments support the view that multiplex base editing to knock out two genes in combination with CRISPR-Cas-mediated targeted integration of a CD19 CAR gene does not negatively impair CAR-T cell function.

### ssDNA HDR template for CRISPR-Cas-mediated targeted integration

**Figure 4:**
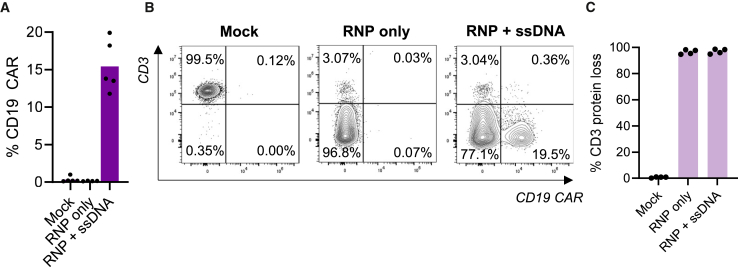
Targeted CD19-CAR transgene integration with ssDNA as HDR template (A) Flow cytometry analysis of targeted integration of the CD19-CAR transgene at the *TRAC* locus in primary human T cells. Cells were electroporated with *TRAC*-targeting RNP complex along with an ssDNA HDR template encoding the anti-CD19 CAR transgene at 100 nM originating from two different ssDNA production batches. (B) Representative flow cytometry plots demonstrating the CAR-TCR-replacement strategy leading to TCR loss upon CAR integration into the *TRAC* locus as measured by loss of CD3 expression. (C) Frequency of cells with CD3 loss following disruption of the TRAC gene. Bars represent means. *N* = 4–5 individual T cell donors.

Using AAV vectors for HDR template delivery is very efficient in many cell types.^63^ However, since AAV vector-related toxicities have been reported in addition to the high manufacturing costs of AAV vectors,^64^^,^^65^ we sought to implement a nonviral integration platform using ssDNA as an HDR template instead of AAV vectors. ssDNA HDR templates with incorporated CTSs consisting of truncated, uncleavable sgRNA protospacer sequences and an NGG PAM on each end of the HDR template have previously demonstrated increased nuclear import of the HDR template and thereby increased integration efficiencies.^47^^,^^49^ From the promoterless anti-CD19 CAR template for targeted integration into the *TRAC* locus we therefore constructed an antisense ssDNA HDR template with incorporated terminal CTS structures by annealing complementary oligonucleotides to each CTS to generate ssDNA with dsDNA CTS ends (Figures S8A and S8B). We performed electroporations of this antisense CAR ssDNA along a *TRAC*-targeting SpCas9 RNP complex to mediate integration in primary human T cells. To further increase integration efficiencies and reduce toxicities of co-electroporation of ssDNA and RNP aggregates, the anionic polymer poly-l-glutamic acid was included during electroporation.^48^^,^^49^^,^^66^ Using this approach, targeted integration of the anti-CD19 CAR was observed in an average of 15% of cells (Figures 4A and 4B). As previously observed, the combined HDR and indels generated at the *TRAC* locus facilitated almost complete knockout of the TCR (97%) (Figures 4B and 4C).

### Nonviral multiplex engineering of allogeneic CAR-T cells

**Figure 5:**
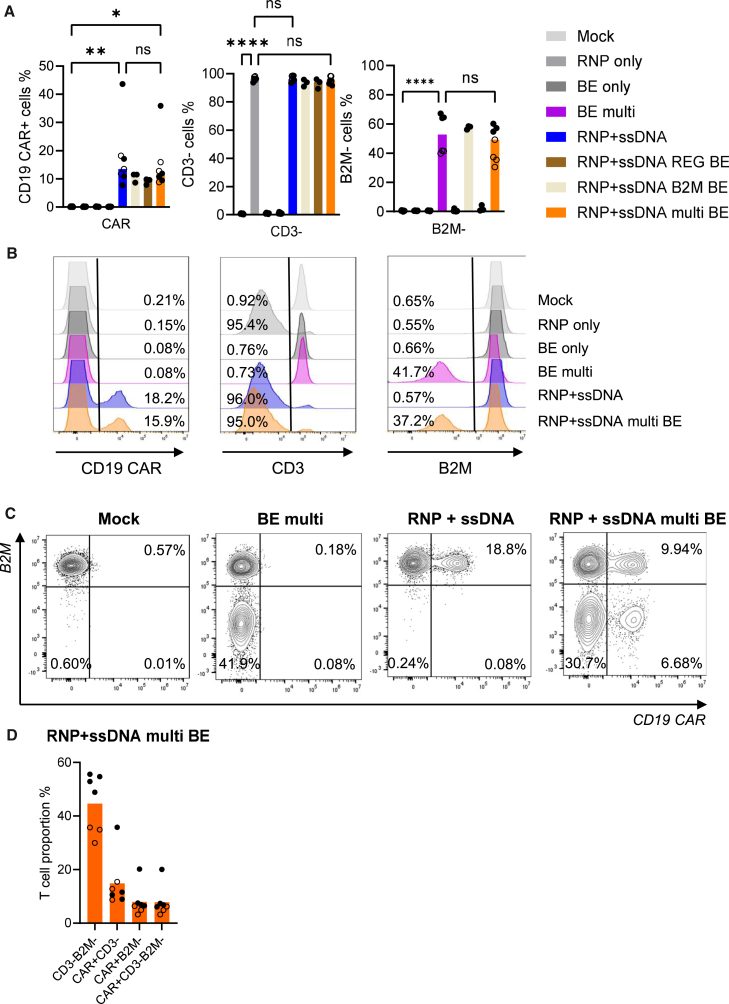
CD19-CAR transgene integration using an ssDNA HDR template combined with multiplex edited CAR-T cells (A) Frequencies of T cells with expression of anti-CD19 CAR, loss of CD3, and loss of B2M 4 days following electroporation of primary human T cells assessed by flow cytometry. The cells were electroporated with indicated combinations of SaCas9 BE mRNA, SaCas9 sgRNAs targeting *B2M* and *REGNASE-1*, SpCas9 RNP complex targeting the *TRAC* locus, and an ssDNA HDR template with homology to the TRAC locus for targeted integration of the CD19 CAR transgene. Empty circles represent delivery of 1 μg of each base editing reagent with 100 nM ssDNA, and full circles represent delivery of 3 μg of each base editing reagent with 150 nM ssDNA. (B) Representative flow cytometry histograms showing CAR integration, CD3 knockout, and B2M knockout. (C) Representative flow cytometry plots showing multiplex editing with simultaneous CAR integration and *B2M* gene knockout. (D) Summary of double and triple editing frequencies based on flow cytometry data. Empty circles represent delivery of 1 μg of each base editing reagent, and full circles represent delivery of 3 μg of each base editing reagent. Bars represent means. Statistical analyses were performed using one-way ANOVA. Significance based on *p* values was determined as follows: ns, not significant; ∗*p* < 0.05; ∗∗*p* < 0.01; ∗∗∗*p* < 0.001; ∗∗∗∗*p* < 0.0001. *N* = 3–7 individual T cell donors.

To engineer allogeneic CAR-T cells with reduced genotoxic risks, nonviral integration using ssDNA as an HDR template and multiplex base editing were combined in a single step. We assessed whether an ssDNA HDR template could achieve multiplex edited CAR-T cells when combined with SaCas9 base editing. Again, multiplex base editing efficiencies were comparable to previous efficiencies and did not interfere with CD19 CAR integration (Figures 5A and 5B). Multiplex editing facilitated knockout of the TCR with high efficiency (95% on average), knockout of B2M (49.2% on average), and CAR transgene integration in an average of 11.9% of cells but as high as 35.9% (Figures 5A and 5B). Overall, this yielded up to 20.2% T cells being CAR^+^B2M^−^ and just as many being CAR^+^B2M^−^TCR^−^ (Figures 5C and 5D). Sanger sequencing confirmed high on-target base editing frequencies at the genomic level for both *B2M* and *REGNASE-1*, and editing frequencies were not affected by simultaneous CD19 CAR integration (Figures S9A and S9B). Again, cell viability was not significantly affected by the multiplex genome editing (Figure S9C).

### Assessment of off-target editing and chromosomal translocations due to multiplex editing

Every gene-editing technology has the potential for editing at off-target sites. Therefore, screening for off-target activity is highly important when considering the clinical potential of gene-edited cellular therapies. For CRISPR-Cas-mediated CAR integration, we used a *TRAC* sgRNA that has previously been thoroughly characterized and shown to have high on-target activity and no detectable off-target activity.^43^^,^^62^ In these studies, off-target sites were predicted by the COSMID algorithm, followed by amplicon sequencing of 40 nominated sites. No detectable indels were observed at these potential off-target sites for CRISPR-Cas9-edited T cells using a high-fidelity Cas9 nuclease, thereby confirming the high specificity of this *TRAC* sgRNA.^43^ Since this is an SpCas9 sgRNA, no sgRNA exchange to the SaCas9 system will occur due to differences in the sgRNA scaffold sequence.

For base editing, an off-target *in silico* analysis was performed using the CRISPOR web tool. Neither of the *B2M* and *REGNASE-1* sgRNAs had any predicted off-target sites with four or fewer mismatches in the 12-bp region adjacent to the PAM (seed region), which is the region most important for sgRNA activity (Figures S10A and S10B). When considering the entire spacer region of the sgRNAs, the *B2M* sgRNA had 3 predicted off-target sites with 4 mismatches, while the *REGNASE-1* sgRNA had 1 predicted off-target site with 3 mismatches and 46 sites with 4 mismatches. For these predicted off-target sites, the majority are intergenic sites, which are less likely to result in adverse effects. Off-target sites in protein-coding exon annotations include the *RPS6KA2* gene for the *B2M* sgRNA and the *ZC3H12C* gene for the *REGNASE-1* sgRNA (Figure S10C). The *RPS6KA2* gene is implicated in controlling cell growth and differentiation. The *ZC3H12C* gene, also referred to as *REGNASE-3*, is a paralog of *REGNASE-1* and demonstrates similar mRNA binding and RNase activity. However, both *RPS6KA2* and *REGNASE-3* gene expression are restricted to the myeloid compartment of the immune system and thus not directly linked to T cell function as assessed by available RNA-seq data (The Human Protein Atlas and Immunological Genome Project). Based on this *in silico* analysis, we consider these two sgRNAs highly specific. Nevertheless, future studies should identify potential off-target sites in an unbiased, empirical fashion with a subsequent assessment of off-target activity and potential functional consequences in T cells.

**Figure 6:**
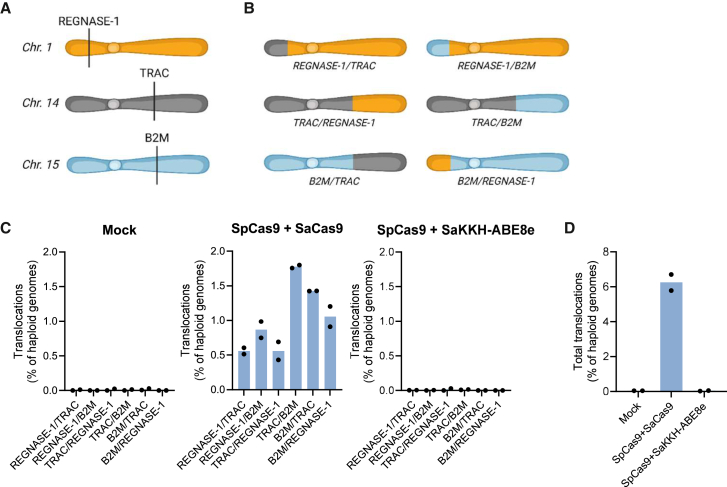
Comparison of the frequency of balanced translocations using SaCas9 or SaKKH-ABE8e in multiplex knockouts (A) Schematic of target gene chromosomal loci with *REGNASE-1* located on chromosome 1, *TRAC* on chromosome 14, and *B2M* on chromosome 15. (B) Schematic representation of the possible balanced translocations that can occur during multiplex editing at the three loci. (C) Assessment of balanced translocations by ddPCR. Frequencies of cells carrying translocations are shown for all six balanced translocations for samples as indicated above each graph. (D) The sum of all balanced translocations detected by ddPCR for individual samples. Bars represent the mean of two individual T cell donors, each analyzed in two technical replicates (*N* = 2 individual T cell donors).

Besides off-target editing, multiplex editing relying on multiple DSBs can result in chromosomal translocations. Because BEs use a Cas9 nickase that only cleaves one DNA strand, we expected that the multiplex SaCas9 base editing approach reduces the occurrence of chromosomal translocations compared to using a regular SaCas9 nuclease, as has previously been demonstrated for SpCas9.^67^ To address this, we established droplet digital PCR (ddPCR) assays to quantify the six balanced translocations that can arise between *TRAC*, *B2M*, and *REGNASE-1* during multiplex editing (Figures 6A and 6B). Primary human T cells were electroporated with *TRAC*-targeting SpCas9 RNP and either SaCas9 active nuclease mRNA + sgRNAs or SaKKH-ABE8e mRNA + sgRNAs. Introduction of on-target indels and A-to-G base conversions were first confirmed with Sanger sequencing (Figures S11A and S11B). ddPCR analyses showed that all 6 balanced translocations were evident in cells where the 2 active nucleases were used simultaneously to edit all 3 loci at frequencies up to 1.8% (Figures 6C and S12). In contrast, when employing base editing for two of the three loci, translocation frequencies were the same as those observed in the mock samples. The additive frequency of all 6 translocations amounted to 6.3% when using active nucleases compared to 0.03% when employing base editing for 2 of the 3 loci (Figure 6D). This 210-fold reduction in translocations demonstrates a significant safety advantage of base editing for multiplex gene knockout in primary human T cells.

## Discussion

Here, we demonstrate a promising strategy for simultaneous multiplex gene knockout and targeted integration to engineer allogeneic CAR-T cells in a single step, while avoiding risks associated with viral HDR template delivery and translocations due to the introduction of multiple DSBs. This nonviral, multiplex genetic engineering strategy simultaneously introduces four genetic changes in the cells (*TRAC*, *B2M*, *REGNASE-1* knockout, and integration of the anti-CD19 CAR transgene) and demonstrates great potential for engineering complex cell and gene therapies. To our knowledge, this is the first platform to describe combined nonviral ssDNA HDR template integration and base editing using orthologous CRISPR-Cas systems based on SpCas9 and SaCas9 in primary human cells in a single step.

Targeted knockout mediated by base editing can be achieved by disrupting a start codon, installing a premature stop codon, or disrupting a splice site. Disrupting a start codon can be efficient; however, the presence of a downstream ATG codon or a less efficient alternative start codon could result in a truncated viable protein.^68^ Hence, careful examination of the downstream sequence would be required. We have designed an sgRNA targeting the B2M start codon for base editing knockout, and no downstream ATG is present in any reading frame in the first exon. Disruption of a splice site has been shown to be the most efficient universal strategy for gene disruption.^69^ However, it is important to consider that this may not be sufficient to create a nonfunctional product. Splice donor disruption has been demonstrated to result in variable outcomes, including NMD, intron retention, or activation of cryptic splice donor sites; regardless of the outcome, all resulted in complete abrogation of protein expression.^37^

Multiplex base editing might reduce the editing efficiency as the increased number of sgRNAs compete for the same BE, which effectively decreases the concentration of functional BEs for a given target locus.^20^ We did not observe reduced efficiencies when combining two sgRNAs in a multiplex setting as previously observed when multiplexing three sgRNAs.^35^ Multiplex base editing of up to four targets also did not show decreases in editing efficiencies upon multiplexing or a decrease in CAR-T cell efficacy.^37^^,^^70^ However, for further increases in the number of targets, the doses and stoichiometries of sgRNAs and BE might need further optimization.

Here, we generate a SaCas9-based all-RNA BE system. SaCas9 sgRNA design is more restricted than it is for SpCas9 due to the increased PAM complexity (5′-NNNRRT-3′ PAM required for SaKKH-ABE8e compared to 5′-NGG-3′ for SpCas9). This, however, increases sgRNA specificity as the frequency of potential off-target sites decreases, potentially making SaCas9-based base editing safer in a clinical context. Further specificity might be conferred by other engineered SaCas9 proteins with increased efficiency and on-target specificity such as SaCas9-HF and enhanced-fidelity SaCas9 (efSaCas9).^71^ *In silico* off-target analysis of the designed sgRNAs in this study did not indicate any prominent off-target sites with a perfect or near-perfect match between the sgRNA and the target sequence in the PAM-proximal region (Figures S10A–S10B). However, further studies should include unbiased experimental tools, particularly within the fully edited CAR^+^CD3^−^B2M^−^ population, to rigorously assess the genome-wide activity of the BEs employed. SaCas9 BEs have also been reported to have a wider editing window compared to SpCas9 BEs, thereby increasing the risk of bystander editing.^55^^,^^72^ When applied for the purpose of gene knockout, this is less of a concern as long as the desired knockout edit is installed. Should SaCas9 fall short for targeting specific genes, other Cas orthologs that might be more suitable are continuously being developed.^73^^,^^74^ A comprehensive study comparing CRISPR-Cas nuclease with adenine base editing for multiplex gene knockout demonstrated several advantages of ABE-edited CAR-T cells.^70^ Here, the authors used lentiviral vectors to deliver the CAR transgene, and the advantages of adenine base editing for knockout included higher manufacturing yields, favorable off-target profile, absence of chromosomal translocations consistent with our findings, enhanced *in vitro* effector functions, increased CAR surface expression, greater proliferative capacity, reduced activation of p53 and DNA damage response pathways, sustained metabolic activity, improved tumor control, and prolonged overall survival in tumor-bearing mice.

Targeted integration and indel formation at the *TRAC* locus enable knockout of the TCR while simultaneously integrating a CAR transgene. This TCR-to-CAR replacement strategy allows for endogenously regulated CAR expression levels and elimination of the TCR to abrogate GvHD.^42^^,^^75^ We achieved 97.4% TCR^−^ cells on average using this strategy. Further depletion of the remaining TCR^+^ cells could be achieved by negative immunomagnetic selection, as previously demonstrated, to obtain a CAR-T cell product purified to have less than 0.05% residual TCR^+^ cells.^43^

We employ HDR-mediated targeted integration using both viral and nonviral HDR templates. Targeted integration of transgenes using ssDNA as HDR template has several advantages over viral delivery methods. The use of AAV6 vectors as HDR template is limited by high manufacturing complexity and cost, p53 activation, and other adverse toxicities.^39^^,^^40^ However, we observed inferior integration frequencies using ssDNA compared to AAV6 vectors, which might be subject to further optimization. Previous studies have demonstrated that designing ssDNA HDR templates with the same sequence as the target strand will increase integration efficiencies as the Cas9 asymmetrically releases DNA strands.^46^^,^^76^^,^^77^ However, this strand bias seems dependent on genomic locus, cell type, and nuclease choice and should therefore be considered carefully.^77^ We recently demonstrated up to 90% knockin efficiency at the CD3ε locus in primary human T cells using a 0.8-kb ssDNA template with CTS ends, highlighting that ssDNA HDR templates can enable remarkably high HDR frequencies and offer a promising nonviral alternative to AAV vectors.^49^ Additional optimization strategies, such as HDR enhancers and enhanced Cas variants could potentially further increase the integration frequencies of large ssDNA HDR templates (>2.5 kb).^78^^,^^79^^,^^80^^,^^81^ Finally, a range of approaches exist to enrich cells expressing the CAR,^82^ but we also note that current release criteria for commercial CAR-T cells are as low as 10% CAR^+^ cells.^83^

Previously, multiplex base editing knockout of two genes has been combined with nonviral integration of a CAR transgene into the *TRAC* locus for simultaneous CAR integration and knockout of the TCR. This study employs Cas12a Ultra and linear dsDNA HDR templates for CAR integration and a SpCas9-derived BE for multiplex knockout achieving efficient multiplex editing (27% CAR integration, 93% TRAC knockout, and 89%–91% base editing knockout of *B2M* and *CIITA*) with a reduction in translocation frequencies to 0.09%.^67^ However, significant DNA-dependent toxicity was observed as reported previously and might in some circumstances be avoided by pharmacological interventions to reduce DNA sensing mechanisms.^66^^,^^84^ ssDNA HDR templates do not pose the same risk of toxicities, and further optimization of strand choice and ssDNA production might achieve higher integration efficiencies, thereby abrogating the need for pharmacological interventions to prevent DNA sensing, which may also pose adverse effects. Our multiplex genome editing approach thus provides an alternative nonviral strategy to avoid limitations observed with dsDNA HDR templates.

For this proof-of-concept study, we selected *B2M* and *REGNASE-1*, two well-characterized genes previously implicated in CAR-T cell engineering, for targeted knockout. Several preclinical and clinical studies have confirmed that *B2M* loss facilitates the production of allogeneic CAR-T cells and allows these cells to evade allogeneic immune rejection.^19^^,^^85^ Similarly, disruption of *REGNASE-1* has previously been shown to enhance CAR-T cell expansion and antitumor activity, primarily in solid tumor models.^56^^,^^57^^,^^59^^,^^86^^,^^87^ One such study demonstrated that CAR-T and TCR T cells with dual disruption of *REGNASE-1* and *Roquin-1* exhibited superior antitumor effects compared to single knockouts.^59^ A recent study using an immunocompetent CD19 CAR-T cell mouse model found that REGNASE-1 knockout alone was insufficient for durable responses, as REGNASE-1-deficient CAR-T cells failed to persist beyond 4 weeks, leading to B cell recovery and a relapse-like phenotype.^87^ Notably, this study showed that combining *REGNASE-1* and *BCOR* knockout improved CAR-T cell persistence and resulted in more sustained tumor control. These findings might explain why *REGNASE-1* knockout did not enhance antitumor efficacy in our study. Importantly, we did not observe any impairment of CAR-T cell function, indicating that multiplex base editing can be applied without compromising CAR-T cell efficacy *in vitro* or *in vivo*. Future work may explore rational combinations of gene knockouts, layering edits that have each been reported to improve CAR-T cell performance.

In conclusion, we combine the use of SpCas9 nuclease and SaCas9 BEs for single-step concurrent genetic knockout and knockin without compromising the functionality of engineered cells *in vitro* or *in vivo*. This non-viral knockin system combined with DSB-free knockout may constitute a safer approach to facilitate complex genetic engineering, and it demonstrates a promising path toward the next wave of cellular therapies.

## Materials and methods

### PBMC and T cell isolation

PB mononuclear cells (PBMCs) were isolated from de-identified buffy coats obtained from healthy adult donors from Aarhus University Hospital Blood Bank by a Ficoll-Paque Plus (Cytiva, Thermo Fisher Scientific) density gradient. Primary human CD3^+^ T cells were subsequently purified by immunomagnetic negative enrichment with the EasySep Human T cell Isolation Kit (STEMCELL Technologies) according to the manufacturer’s instructions.

### Cell lines and cell culture

K562 cells were cultured at a density of 2–4E5 cells/mL in RPMI 1640 media (Lonza) supplemented with 10% fetal bovine serum (FBS; Sigma-Aldrich), 100 U/mL penicillin and 100 μg/mL streptomycin (Gibco), and 2 mM l-glutamine (Lonza). Viral HEK293F production cells were cultured in Viral Production Medium (Gibco, Thermo Fisher) according to the manufacturer’s instructions at a density of 4–6E6 cells/mL on an orbital shaker incubator at 37°C and 8% CO_2_ in a humidified incubator. Primary human T cells were cultured in X-vivo 15 medium (Lonza) supplemented with 5% human serum (Sigma-Aldrich), 21.8 IU/mL interleukin-2 (IL-2; PeproTech), and 10 ng/mL IL-7 (PeproTech). T cells were activated for 3 days with Human T-Activator CD3/CD28 Dynabeads (Thermo Fisher Scientific) at a 1:1 cell-to-bead ratio. When needed, cells were reactivated for another 3 days at 7–10 days post-removal of the initial activation beads. At reactivation, the culture medium was supplemented with 10 ng/mL IL-15 (PeproTech). T cells were cultured at a density of 1E6 cells/mL. K562 cells and T cells were cultured at 37° and 5% CO_2_ in a humidified incubator.

### sgRNA design for SaCas9 base editing

All sgRNAs for SaCas9 base editing-mediated knockout of *B2M* and *REGNASE-1* genes were designed using the CRISPR RGEN BE-Designer web tool.^60^ Outputs were screened for sgRNAs that would either target the start codon, create a premature stop codon, or disrupt a splice site. Emphasis was put on sgRNAs targeting exons 1–4. sgRNA designs were ordered as oligos with overhangs for cloning into a pX330-derived plasmid previously described,^50^ in which the cytomegalovirus-Cas9 expression cassette is eliminated. Furthermore, the SpCas9 scaffold has been replaced with that of SaCas9. The most promising ABE sgRNAs were ordered as synthetically modified sgRNAs with the three terminal nucleotides in each end modified by 2′-*O*-methyl 3′-phosphorothioate (SBS Genetech or Synthego).^88^ All sgRNA sequences can be found in Tables S1 and S2.

### Plasmids

Plasmid expressing SaKKH-ABE8e (Addgene plasmid no. 138502) was a gift from David Liu and provided by Addgene. The ABE SaKKH-ABE8e was cloned into an IVT-suitable plasmid containing a T7 promoter with an AG initiator, allowing cotranscriptional capping with CleanCap reagent AG from TriLink. The stop codon of the BE is followed by a 93-bp 3′ UTR of the murine *Hba-a1* gene, then a 50-bp-long poly(A), and finally a unique restriction site.

Plasmid expressing SaCas9 (pX601, a gift from Feng Zhang, Addgene plasmid no. 61591) was used as template for PCR (Platinum SuperFi II Green PCR Master Mix, Thermo Fisher), with a forward primer adding a T7 promoter with an AG initiator (5′-TAATACGACTCACTATAAGGGAGAGCCGCCACCATGGCCCCAAAGA-3′) and a reverse primer adding a 50-bp-long poly(A) (5′-poly(T)_50_GAGGCTGATCAGCGAGCTCTAG-3′). The PCR amplicon was verified on a 1% agarose gel and extracted from the gel (GeneJET Gel Extraction Kit, Thermo Fisher), followed by IVT.

### IVT of SaKKH-ABE8e and SaCas9 mRNA

The SaKKH-ABE8e and SaCas9 IVT plasmids were linearized with a unique restriction enzyme immediately downstream of the poly(A), allowing a T7 RNA polymerase run-off IVT process. Successful linearization was verified by gel electrophoresis, and the linearized plasmid was precipitated with 5 M ammonium acetate and ethanol prior to IVT. IVT was performed using the MEGAscript T7 kit (Invitrogen) with uridine substituted with N1-methylpseudouridine (Trilink Biotechnologies) and cotranscriptional capping with CleanCap AG (Trilink Biotechnologies) at a 0.8:1 CleanCap-to-GTP ratio. The IVT mRNA was purified and concentrated using the RNA Clean & Concentrator Kit (Zymo Research) according to the manufacturer’s instructions. mRNA integrity was verified on a Bioanalyzer 2100 (Agilent) and quantified by UV-visible light (UV-vis) spectrophotometry. All mRNA was stored at −80°C.

### Recombinant AAV6 vector production

An anti-CD19 CAR transgene (comprising a granulocyte-macrophage colony-stimulating factor receptor-α leader sequence, the FMC63 single-chain variable fragment [scFv], CD8a hinge and transmembrane sequences, a 4-1BB co-stimulatory domain, and the CD3ξ intracellular domain) was cloned into the plasmid AAV-multiple cloning site plasmid (Agilent Technologies) containing AAV2 ITRs applicable for AAV vector production. AAV6 vectors were produced as described previously, with few modifications.^63^ Viral HEK293F production cells from the AAV-MAX Helper-Free AAV Production System Kit (Gibco, Thermo Fisher) were handled according to the manufacturer’s instructions and seeded at a density of 3E6 cells/mL. Viral production cells were transfected using a mixture of OptiMEM (Gibco, Thermo Fisher), 5 mM sodium butyrate, and polyethylenimine MAX (PEI MAX) (Polysciences) with a PEI MAX to DNA ratio of 3:1 (540 μL PEI MAX and 180 μg total DNA for a 250-mL production) with 1.5 μg total plasmid DNA per mL transfection culture at a 1:3.5 ratio between the ITR-containing plasmid and the pDGM6 packaging plasmid containing the AAV6 cap genes, AAV2 rep genes, and adenovirus helper genes required for AAV vector production (Addgene plasmid no. 110660, a gift from David Russell). The transfection mix constitutes ∼13.33% of the culture volume and was incubated for 15 min at room temperature before being added to the production cells. Cells were harvested 72 h post-transfection and lysed by three freeze-thaw cycles followed by a 45-min incubation with 200 U/mL Turbonuclease (Sigma-Aldrich). AAV vectors were purified on an iodixanol density gradient (15%–25%–40%–58% iodixanol solutions prepared from Optiprep 60 [STEMCELL Technologies]) by ultracentrifugation at 48,000 rpm for 2 h at 18°C. AAV vectors were extracted at the 40%–58% iodixanol interface, diluted with 1× sorbitol containing 0.001% pluronic acid, and concentrated using Amicon Ultra-15 centrifugal filters (Merck). AAV6 vectors were aliquoted and stored at −80°C. AAV6 vectors were titered using two-dimensional ddPCR to determine the number of viral vector genomes, as described previously.^89^ The ITR primers and probes used for ddPCR were as follows: ITR forward: 5′-GGAACCCCTAGTGATGGAGTT-3′; ITR reverse: 5′-CGGCCTCAGTGAGCGA-3′; ITR probe, 5′-FAM-CACTCCCTCTCTGCGCGCTCG-IBFQ-3′ (IDT). The bovine growth hormone polyadenylation signal (bGH polyA) primers and probe used for ddPCR were as follows: bGH forward: 5′-GCCAGCCATCTGTTGT-3′; bGH reverse: 5′-GGAGTGGCACCTTCCA-3′; bGH probe, 5′-HEX-TCCCCCGTGCCTTCCTTGACC-IBFQ-3′ (IDT).

### Generation of ssDNA HDR templates

ssDNA containing the same HDR donor as was used for AAV6 vectors described above but carrying additional CTSs was produced using the Guide-it Long ssDNA Production System version 2 (Takara). Briefly, the dsDNA template was amplified from the AAV6 transfer plasmid with appropriate phosphorylated primers (one phosphorylated primer and one non-modified primer). The amplified dsDNA template was purified, followed by the Strandase reaction to digest the phosphorylated strand (sense strand), leaving the other strand (antisense strand) as ssDNA. Both dsDNA and ssDNA were purified using magnetic-based purification (HighPrep PCR Clean-up magnetic beads, MagBio Genomics). The ssDNA was precipitated and resuspended with RNAse/DNAse-free H_2_O to increase the concentration before electroporation and quantified by UV-vis spectrophotometry. ssDNA was verified by comparison with its dsDNA template by gel electrophoresis. To create dsDNA ends in the ssDNA HDR template, CTS oligos were mixed with the ssDNA HDR template at a 6:1 oligo-to-ssDNA ratio, followed by incubation at 94°C for 2 min, 70°C for 1 min, and room temperature for 15 min. Annealed ssDNA HDR templates were stored at −20°C. Primer and oligo sequences for dsDNA and ssDNA generation and for CTS annealing are supplied in Table S4.

### Electroporation

K562 cells and primary human T cells were electroporated using the Lonza 4D-Nucleofector device (Core and X unit) in a 20-μL Nucleocuvette strip format for electroporation of 4E5-1E6 cells as previously described.^90^ Harvested cells were washed in PBS, resuspended in an appropriate electroporation buffer, and desired gene editing reagents added before electroporation. The exposure time to the electroporation buffer was kept to a minimum. Cells were electroporated using the following electroporation programs and buffers: K562 cells (P3-CM138, OptiMEM, Gibco) and primary human T cells (P3-EO115 for following AAV transduction and P3-EH115 for ssDNA transfection, P3 buffer, Lonza). T cells were electroporated 4 days after activation and thawing. RNP complexes were formed by mixing recombinant Cas9 protein (Alt-R S.p. Cas9 Nuclease V3, IDT) and a synthetically modified sgRNA (Synthego) at a molar ratio of 1:2.5 followed by incubation at 25°C for 15 min. RNP was mixed with cells and electroporation buffer yielding a final concentration of 300 μg/mL Cas9 protein and 160 μg/mL sgRNA. For base editing conditions, the BE mRNA and each of the BE sgRNAs were added at 1 or 3 μg as indicated. For plasmid-based delivery to screen BE sgRNAs, 1 μg of each plasmid component was added. For HDR, the ssDNA HDR template was added at 100 or 150 nM as indicated. For AAV HDR template delivery, cells were transduced with rAAV6 vectors within 15 min post-electroporation at an MOI of 10,000. Medium was changed 16–18 h following AAV transduction to remove excess AAV vectors and limit related toxicities.

### Flow cytometry

Flow cytometry analysis was used to determine viability and gene editing efficiency by protein expression readout 4–6 days following electroporation. Flow cytometry was performed on a 4-laser Cytoflex S flow cytometer (Beckman Coulter) using 96-well V-bottom plates. Approximately 2E5-5E5 cells were centrifuged at 300 × *g* for 5 min, washed in PBS, and resuspended in staining buffer (PBS, 2% FBS, 2 mM EDTA). Cells were stained with fluorochrome-conjugated antibodies and a viability dye for 30 min and washed twice in staining buffer. Lastly, cells were resuspended in 100 μL staining buffer or fixation buffer (0.9% formaldehyde), and fluorescence was analyzed with the CytExpert acquisition software. If required, stained beads were included for compensation (UltraComp eBeads and ArC Amine Reactive Compensation Bead Kit, both from Invitrogen). The antibodies used are listed in Table S7. Data analysis was performed using FlowJo version 10.8/9 software (BD Life Sciences) with the gating strategy depicted in Figures S13 and S14.

### Proliferation assessment by flow cytometry cell counting

T cell proliferation was determined at 4 and 13 days after electroporation using CountBright Absolute Counting Beads (Thermo Fisher Scientific) according to the manufacturer’s instructions, with slight modifications. Briefly, a 40-μL cell suspension containing primary human T cells was mixed with 5 μL counting beads and 2.5 μL of 50 μg/mL propidium iodide and adjusted to a total volume of 100 μL with fluorescence-activated cell sorting FACS buffer (PBS, 2% FBS, and 2 mM EDTA). Cells were incubated for 5 min at room temperature in the dark and analyzed on a 4-laser Cytoflex S flow cytometer (Beckman Coulter) with a fixed stop condition at 60 μL using the CytExpert acquisition software. The live cell concentrations were calculated as follows: live cells/mL = (number of live cells counted/number of beads counted) × (number of beads added to the sample/sample volume). Live cell concentrations were used to determine the total cell number based on the total culture volume. Data analysis was performed using FlowJo version 10.8/9 software (BD Life Sciences).

### Phenotype and exhaustion analysis by flow cytometry

To assess phenotypic characteristics and exhaustion, status cells were stained with four distinct antibody panels and analyzed using a 4-laser CytoFlex S flow cytometer (Beckman Coulter). The panels were designed to investigate (1) CAR expression and CD3/B2M knockout, (2) differentiation, (3) activation, and (4) exhaustion. The following antibody panels were utilized: panel 1 (FMC63-scFv, CD3, and B2M), panel 2 (FMC63-scFV, CCR7, and CD45RA), panel 3 (FMC63-scFv, CD69, CD25, and HLA-DR), and panel 4 (FMC63-scFv, PD-1, and LAG-3). Specific antibodies are listed in Table S7. CD19 CAR expression was assessed 5 days post-electroporation using panel 1. On day 8 post-electroporation, cells were stained with panels 2, 3, and 4. Prior to antibody staining, all cells were incubated with Human TruStain FcX (BioLegend, catalog no. 422301) for 15 min to block non-specific binding to Fc receptors. Following antibody staining, cells were fixed in PBS containing 0.99% formaldehyde for 15 min. Viability dyes were included to exclude dead cells from the analysis. Flow cytometry data were gated using unstained controls, single-stained controls, and fluorescence minus one controls to ensure accurate interpretation of marker expression. Data were analyzed using FlowJo version 10.10, and graphs were generated with GraphPad Prism version 10.

### Sanger sequencing and quantification of indels and base editing efficiency

To quantify indel or base editing efficiencies, genomic DNA was extracted using the QuickExtract solution (Nordic Biosite). Primers for PCR amplification (Phusion Green Hot Start II High-Fidelity PCR Master Mix or Platinum SuperFi II Green PCR Master Mix, both from Thermo Fisher) were designed to amplify a 400- to 600-bp fragment surrounding the protospacer at the *TRAC*, *B2M*, and *REGNASE-1* loci. Primers are listed in Table S3. PCR amplification products were verified by gel electrophoresis and purified from the gel using the GeneJET Gel Extraction kit (Thermo Fisher). Sanger sequencing was performed commercially (Eurofins). Indel frequencies were quantified using the Inference of CRISPR Edits (ICE) analysis web tool (Synthego). Base editing efficiencies were quantified using the BEAT.^61^

### Amplicon sequencing

Genomic DNA was extracted using the QuickExtract solution (Nordic Biosite) 4 days after electroporation. An initial PCR (Platinum SuperFi II Green PCR Master Mix, Thermo Fisher) was performed to amplify the *B2M* and *REGNASE-1* genomic loci and attach TruSeq adapters. All primers are listed in Table S5. Primer pairs without adapter sequences were used to verify the PCR before the *B2M* and *REGNASE-1* genomic loci were amplified using primers with TruSeq adapters. PCR products were purified using HighPrep PCR Clean-up magnetic beads (MagBio Genomics) according to the manufacturer’s instructions and the concentration was measured using a Qubit Flex Fluorometer (Invitrogen, Thermo Fisher Scientific). A second PCR was performed using 4 ng from the first PCR and index primers and subsequently purified using HighPrep PCR Clean-up magnetic beads (MagBio Genomics) according to the manufacturer’s instructions, followed by library pooling and dilution to a final library concentration of 55 pM. The library was spiked with diluted PhiX control (Illumina), and 20 μL of the final sample was sequenced with 150-bp-long paired end reads by an Illumina iSeq 100 instrument (Illumina). Editing rates were analyzed using CRISPresso2 software^91^ with the following settings: min_frequency_alleles_around_cut_to_plot = 0.01, plot_window_size = 25, quantification_window_size = 11, and quantification_window_center = −10.

### Bulk RNA-seq

Abnormal transcript isoforms harboring premature stop codons resulting from base editing-mediated disruption of splice sites underwent degradation through NMD. To allow detection of these transcripts, cells were treated with 100 μg/mL cycloheximide (Santa Cruz Biotechnology) for 8 h prior to RNA extraction to inhibit NMD. RNA was extracted using the ReliaPrep RNA Cell Miniprep system (Promega) according to the manufacturer’s instructions. Acceptable quality of RNA was confirmed using the Agilent 2100 Bioanalyzer before samples were submitted to Novogene for RNA-seq. mRNA was enriched from total RNA using poly(T) oligo-attached magnetic beads. Following fragmentation, first-strand cDNA synthesis was performed using random hexamer primers, followed by second-strand synthesis. Library preparation included end repair, A-tailing, adapter ligation, size selection, PCR amplification, and purification. Libraries were quantified using Qubit and real-time PCR, and size distribution was confirmed using a bioanalyzer. Pooled libraries were sequenced on an Illumina NovaSeq X Plus platform, yielding a minimum of 56 million raw reads per sample. FASTQ files from paired-end sequencing were adaptor trimmed using Trim Galore 0.6.7. Mapping to the human reference genome Hg38/Ensembl 88 was performed with STAR 2.7.10a using two-pass mapping with a shared splice junction database across the samples and a requirement of a 3-bp overhang across known splice junctions in the second round of mapping for spliced alignments. To identify intron retention, reads were split into intronic and exonic parts using gatk 4.2.0.0 and the SplitNCigarReads function, and a 3-bp overhang of intron sequence at both sides of the alternative splice donor site in intron 4 was required to call intron retention. The mapped reads were analyzed in the Integrative Genomics Viewer 2.19.1.

### Cytotoxicity assay

To assess the cytotoxic potential of CAR-T cells, a flow cytometry-based killing assay was performed using a GFP-expressing NALM6 leukemia cell line (NALM6-GFP) as target cells. Target cells were seeded at a density of 20,000 cells per well in a 96-well round-bottom plate and co-cultured with CAR-T cells or mock electroporated T cells at various E:T ratios. All conditions were performed in technical duplicates using CAR-T cells generated from two independent T cell donors (biological duplicates). Controls included target cells cultured alone (target only), and effector cells cultured without target cells (effector only). Co-cultures were incubated at 37°C and 5% CO_2_ for 24 h. Following incubation, cells were stained with 7-aminoactinomycin D (BioLegend) to exclude non-viable cells and analyzed using an Agilent NovoCyte Flow Cytometer. The absolute counting feature of the NovoCyte software was used to quantify the number of live GFP^+^ target cells in each condition. Specific killing was calculated based on the reduction in live GFP^+^ target cells in CAR-T cell conditions relative to the mock T cell control. In cases where this calculation yielded negative values—in other words, when more live GFP^+^ target cells were detected in the CAR-T cell condition than in the mock control—these were interpreted as zero killing and plotted accordingly.

### ELISA

To quantify cytokine secretion following target cell engagement, levels of TNF-α and IFN-γ were measured in supernatants collected from 24 h cytotoxicity assays. Supernatants were harvested from the E:T ratio of 5:1 across all experimental conditions from two independent T cell donors (biological duplicates). Samples were stored at −20°C until analysis. ELISAs were performed using the Human TNF-α and Human IFN-γ ELISA MAX Deluxe kits (BioLegend) according to the manufacturer’s instructions. Briefly, 96-well ELISA plates were coated with capture antibody, blocked, and incubated with appropriately diluted samples at 4°C overnight. Sample dilutions were determined in advance based on a preliminary dilution series to ensure that absorbance values fell within the linear range of the standard curve. Following the final incubation with streptavidin-horseradish peroxidase, 3,3′,5,5′ tetramethylbenzidine substrate was added to initiate the colorimetric reaction. After sufficient color development, the reaction was stopped with 2 N H_2_SO_4_, and absorbance was measured at 450 nm with reference correction at 570 nm using a SpectraMax i3x microplate reader (Molecular Devices). Final absorbance values were calculated by subtracting the 570-nm reading from the 450-nm reading to correct for background signal. Cytokine concentrations were calculated based on the corresponding standard curve generated from the recombinant standards provided in the kit. All samples were analyzed in technical triplicates for each donor.

### *In vivo* xenograft mouse model

Animal experiments were approved by and conducted in accordance with the accepted protocol (2023-15-0201-01407) from the Danish Animal Inspectorate. Eight-week-old NOG mice (NOD.Cg-Prkdc^scid^Il2rg^tm1Sug^/JicTac) were obtained from Taconic. The xenograft models were established by intravenous injection of 5E5 NALM6 cells resuspended in 200 μL sterile PBS. The NALM6 cells were previously lentivirally transduced and FACS sorted to generate a cell line with constitutive expression of GFP-NLuc. Three days later, baseline tumor engraftment was assessed by bioluminescence imaging using the Newton 7.0 (Viber). Four days post-NALM6 inoculation, mice were randomized into 3 groups: 4 mice receiving mock-electroporated T cells, 8 mice receiving unmodified CAR-T cells, and 8 mice receiving BE-modified CAR-T cells. We administered via tail vein injection 5E5 cells in 200 μL sterile PBS. Unfortunately, two mice died during the initial scanning due to isoflurane intoxication and were therefore excluded from further analysis, as their deaths were not deemed to be treatment related. Mice were monitored biweekly by bioluminescence imaging using the Newton 7.0 (Viber) system to assess tumor burden and treatment efficiency. Each scan was performed 5–10 min after intraperitoneal injection of 0.04 μmol Nano-Glo Fluorofurimazine In Vivo Substrate (Promega, catalog no. N4100) under isoflurane anesthesia. Exposure time varied based on tumor signal, ranging from 30 s to 1.5 min. Background luminescence was subtracted from each measurement. Imaging data were analyzed using Kuant software and total photons per second steradian (ph/s/sr) per mouse were quantified. In addition, mice were monitored at least three times per week for weight changes and signs of treatment/tumor-related toxicity, including body weight loss exceeding 20%, fur loss, excessive spinal curvature, or hindlimb paralysis. Mice were euthanized by cervical dislocation upon exhibiting any of these symptoms and/or a bioluminescence signal exceeding 1E9 ph/s/sr (total minus background). On the day of termination, blood was collected via retro-orbital bleeding, spleens were dissected and mechanically dissociated through a 70-μm cell strainer, and bone marrow was harvested and aspirated using a 27G needle under sterile conditions. Samples were stored either in PBS supplemented with 10 mM EDTA or in RPMI containing DNase and heparin until further analysis.

### Translocation quantification by ddPCR

ddPCR assays were designed to quantify balanced translocations between the *TRAC*, *B2M*, and *REGNASE-1* loci. A ddPCR assay for the *TERT* gene was used as reference. We used 50 ng FastDigest HindIII (2–5 U/reaction) (Thermo Fisher) digested gDNA as template for a 20-μL PCR reaction with 1 μL of a 20× PrimeTime qPCR Assay primer/probe mix, 1 μL (5 μM) 20× TERT assay reference probe (HEX), 10 μL 2× ddPCR Supermix for Probes (no deoxyuridine triphosphate), and nuclease-free water. Droplets were generated with the QX200 Droplet Generator using 20 μL sample and 70 μL Droplet Generation Oil in a DG8 cartridge covered by DG8 Gaskets for QX200 Droplet Generator. After droplet generation, droplets were gently pipetted into a 96-well PCR plate and sealed using a PX1 PCR Plate Sealer and pierceable foil heat seal at 185°C for 5 s. PCR was run as follows: 95°C for 10 min; 45 cycles of 94°C for 30 s, 60°C for 1 min, and 72°C for 2 min; 98°C for 10 min; and hold at 12°C. All ramp rates were adjusted to 2°C/s. All primers and probes were ordered from IDT and the sequences are listed in Table S6, while all other unspecified devices and reagents were acquired from BioRad. Data analysis was performed using QuantaSoft Analysis Pro software (BioRad).

### Statistical analysis

Is the data are presented as means with individual data points shown. Unpaired Student’s t tests (two tailed) and one-way ANOVA were performed as indicated. Significance based on *p* values was determined as follows: ns, not significant; ∗*p* < 0.05; ∗∗*p* < 0.01; ∗∗∗*p* < 0.001; ∗∗∗∗*p* < 0.0001. Statistical analysis and graphing were conducted using GraphPad Prism 10 software.

## Data availability

The datasets supporting the conclusions of this article are included within the article and its supplemental information. All data presented in this study are available from the corresponding author upon reasonable request.

## Acknowledgments

We would like to thank Jonas Holst Wolff and Thomas Wisbech Skov for help with the NGS experiments and Pernille Thornild Møller and Tania Toft Haugdal for technical assistance. The schematic figures were created using Biorender.com. N.S.M. was supported by a grant from the 10.13039/501100009708Novo Nordisk Foundation (NNF19OC0058238) and institutional support from the Department of Biomedicine, 10.13039/100007605Aarhus University. In addition, this project was funded by the 10.13039/501100000780European Union under grant agreement no. 101057438. The views and opinions expressed here are those of the authors only and do not necessarily reflect those of the European Union or the European Health and Digital Executive Agency. Neither the 10.13039/501100000780European Union nor the granting authority can be held responsible for them. M.G.P. was supported by a grant from Landmand af Ølufgård Peder Nielsen Kristensens Mindefond. Funding in the Bak lab is supported by a grant from the Danish Health Authority (4-1612-391/1), the EU Commission in the form of an ERC starting grant (project 101041231, Horizon Europe Pillar I), a Lundbeck Foundation fellowship (R238-2016-3349), the Independent Research Fund Denmark (0134-00113B, 0242-00009B, and 9144-00001B), an AIAS-COFUND (Marie Curie) fellowship from the 10.13039/501100009388Aarhus Institute of Advanced Studies (AIAS) co-funded by Aarhus University’s Research Foundation and the European Union’s Seventh Framework Program under grant agreement 609033, the 10.13039/501100009708Novo Nordisk Foundation (NNF17OC0028894), Innovation Fund Denmark (8056-00010B), the 10.13039/501100002808Carlsberg Foundation (CF20-0424 and CF17-0129), Slagtermester Max Wørzner og Hustru Inger Wørzners Mindelegat, the A.P. Møller Foundation, the Riisfort Foundation, the Agnes and Poul Friis Foundation, and a Genome Engineer Innovation Grant from Synthego.

## Author contributions

N.S.M. and R.O.B. conceived the study and designed the experiments. N.S.M., S.R., A.D.B., S.F.S., M.G.P., H.F., and M.K.T. performed the experiments. Data analyses and interpretations were performed by N.S.M., S.R., A.D.B., S.F.S., T.T., and R.O.B. All experiments were supervised by R.O.B. N.S.M. took the lead in writing the manuscript, with input from all authors.

## Declaration of interests

R.O.B. holds equity in Kamau Therapeutics and UNIKUM Therapeutics and is a cofounder of UNIKUM Therapeutics. Neither company was involved in the present study. R.O.B. is listed as an inventor on patents and patent applications related to CRISPR-Cas and cellular therapies. R.O.B. reports research funding from Novo Nordisk.

## Supplemental information

Document S1. Figures S1–S14 and Tables S1–S7

Document S2. Article plus supplemental information
