# Supplementary material for: Orthogonal CRISPR systems for targeted integration and multiplex base editing enable nonviral engineering of allogeneic CAR-T cells
Source: Mol Ther. 2025 Aug 26;33(12):6082–100. doi: 10.1016/j.ymthe.2025.08.032 (PMC12703164; doi:10.1016/j.ymthe.2025.08.032)
Supplement: Document S1. Figures S1–S14 and Tables S1–S7 [file mmc1.pdf]

YMTHE, Volume *33*

Supplemental Information

Orthogonal CRISPR systems for targeted integration and multiplex base editing enable nonviral engineering of allogeneic CAR-T cells

Nanna S. Mikkelsen, Sujan Ravendran, Amalie D. Broksø, Sigrid Fu Skjelbostad, Maya G. Pedersen, Hongyu Fang, Thorkild Terkelsen, Martin Kristian Thomsen, and Rasmus O. Bak

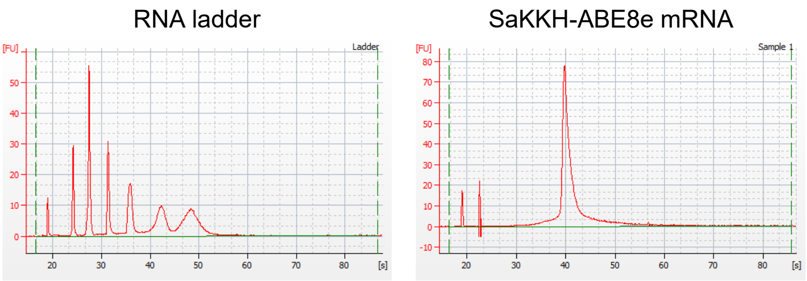

FIGURE S1: Verification of IVT mRNA integrity and quality. IVT mRNA production of the SaKKH-ABE8e adenine base editor verified on the Bioanalyzer 2100.

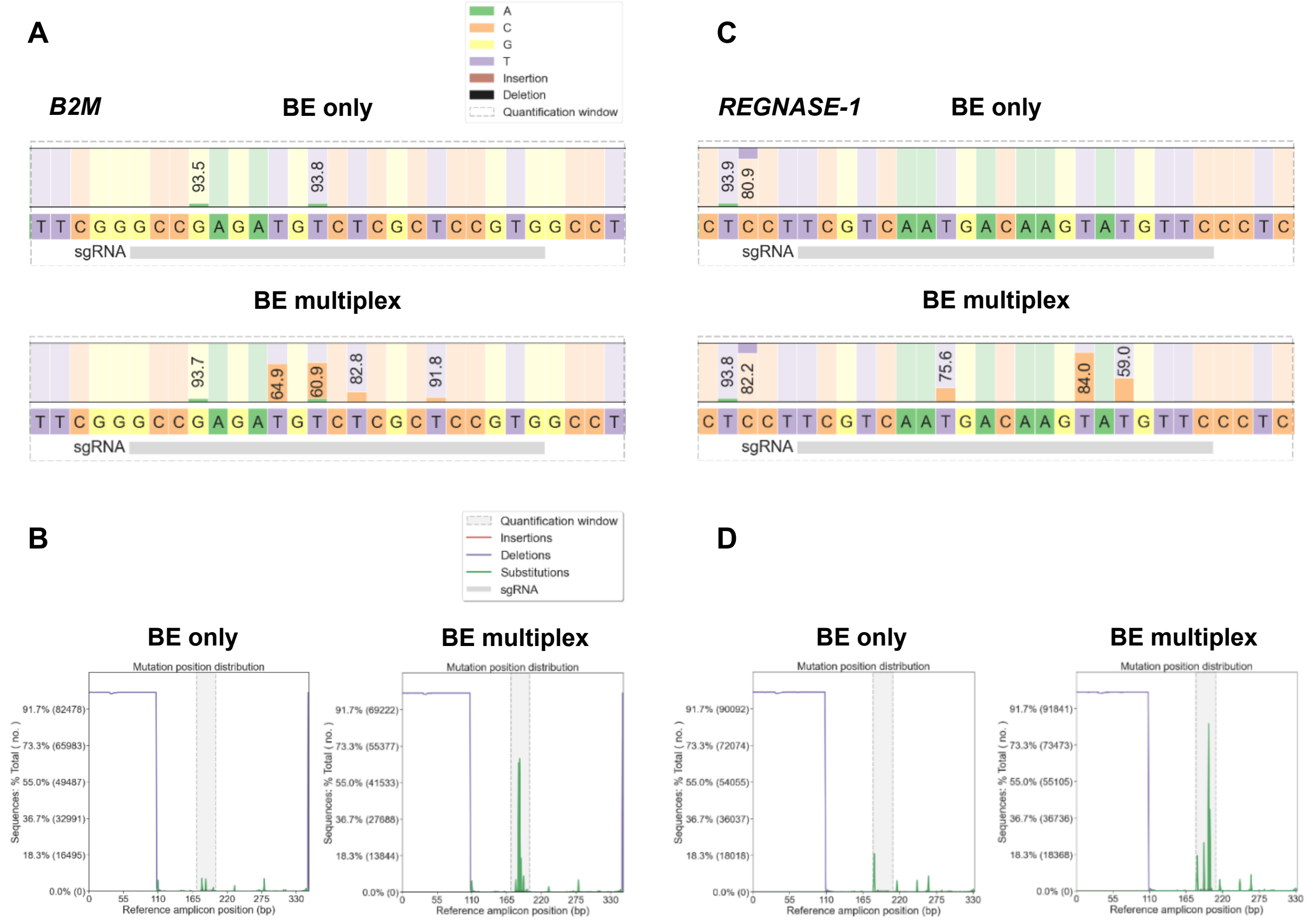

FIGURE S2: NGS CRISPResso2 results of B2M and REGNASE-1 base editing. Representative CRISPResso2 results from NGS analyses demonstrating base editing frequencies as well as characterization of all modifications including substitutions and INDELs at the **(A, B)** B2M and **(C, D)** REGNASE-1 locus. Small peaks are observed in the BE only samples due to sequencing artifacts as no editing can occur in these control samples. BE=base editor. Representative from N=4 individual T cell donors.

A

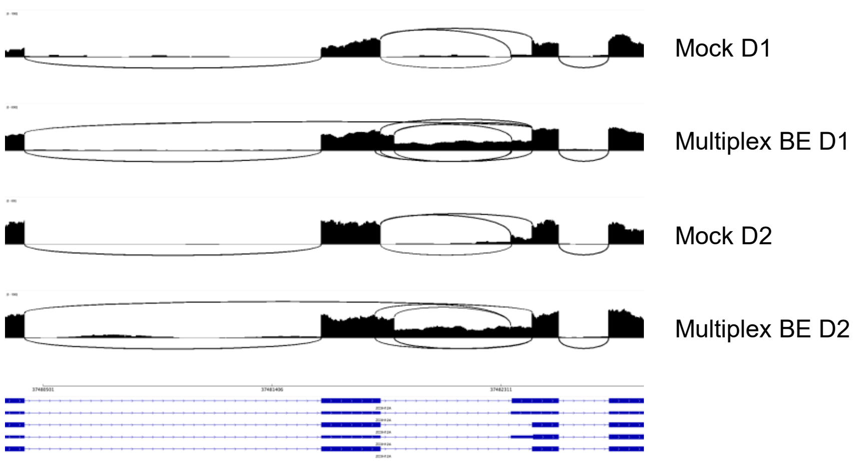

B

Outcome	RNA level	Protein level
Exon 4 skipping (ES)	NC_000001.11(NM_025079.3):r.584_818del	NC_000001.11(NP_079355.2):p.(Asp195Glyfs*96) Resulting in disruption of the reading frame and assumes loss-of-function as most of the protein is encoded by exon 4-6.
Alternative 5'SS in exon 4 (5'SS_-21)	NC_000001.11(NM_025079.3):r.798_818del	NC_000001.11(NP_079355.2):p.(Tyr267_Lys273del) Resulting in a 7aa deletion in the highly conserved RNase domain spanning amino acids 112-297.
Alternative 5'SS in intron 4 (5'SS_+55)	NC_000001.11(NM_025079.3):r.818_819ins818+1_818+55 (*)	NC_000001.11(NP_079355.2):p.(Phe274Tyrfs*8) Resulting in the presence of a premature stop codon positioned 8 codons inside intron 4.
Intron 4 retention (IR)	NC_000001.11(NM_025079.3):r.818_819ins818+1_819-1 (**)	NC_000001.11(NP_079355.2):p.(Phe274Tyrfs*8) Resulting in the presence of a premature stop codon positioned 8 codons inside intron 4.

* Corresponds to inserting the following sequence in the RNA:
NC_000001.11(NM_025079.3):r.818_819insGTATGTTCCCTCCCAGAGGCCCTGACAGACTTGGGGTCCACAGGGGAAGCCAGAG

** Corresponds to inserting the following sequence in the RNA:
NC_000001.11(NM_025079.3):r.818_819insGTATGTTCCCTCCCAGAGGCCCTGACAGACTTGGGGTCCACAGGGGAAGCCAGAGGTGCCCTTGGC
AAGGGTGGAGCTGGGGGCTGGGCTCTGCGGGGCCCTGTGGCCATGGGAGGTTGCGGGTCTTGGCTCCAGGCAGCTTTGAGAGTGAGACGGATA
GCTCACCACATAGGAGAAATCAGACCGGGACCAGGCAGGCTGTGGGGTGGAGAGAGTGGCTAATTTGGGAGATAGAGCCGTAGCACTTATGA
GGGGATGTATGTGGTTGATGGTTCCAGGTGGCCTCTCTACGAACCAACATGGCATCTCTCGAGCAGAGGCCATGGGCCAGTGGGTGCGGGCTG
CCATCCCCCGACGACCTCAGAGAGGGAGTTCCCCTAAAGGTGCCCATGGGCTGTGGCCCTCTAGACCGGGGATCCCTTTCTCCTCCCTTGGTATG
ATCCCACCCATAGAGCAGTTTTGCGGGGTCCTGGACAGGCCCCAGTTTTAGGTGTCACTCTCCTATTCTTCCCAGCAAGGCTTGCCATCGGCCCC
TTCTCAGCAATTAGAAGTCCCTGCATGGCTCCCACCTCCAGAGAGTTCTTTGCACCCTCTGCAG

C

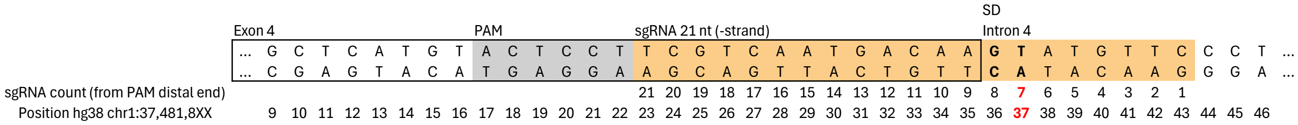

FIGURE S3: Bulk RNA-seq assessment of abnormal splice outcomes following base editing to disrupt the canonical splice site for exon 4 for REGNASE-1. (A) Sashimi plot demonstrating different splice patterns for mock edited and base edited samples for two independent primary human T cells donors (D1 = donor 1, D2 = donor 2). **(B)** Outcomes following disruption of the splice donor site by base editing described by HGVS nomenclature. Genomic positions are based on the MANE reference transcript. **(C)** Schematic figure depicting the intersection between exon 4 and intron 4 to demonstrate on-target and bystander base edits in transcripts with nucleotide locations marked both based on the ABE sgRNA spacer sequence position and the genomic position.

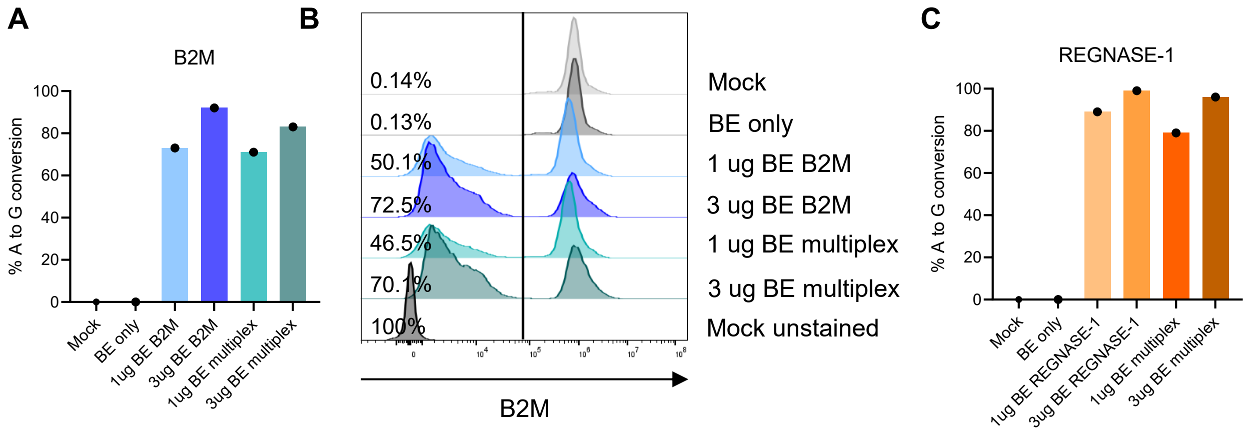

FIGURE S4: Comparison of base editing efficiency with increased amounts of base editing reagents in primary human T cells. Cells were electroporated with 1 or 3 ug of the indicated base editing reagents (base editor mRNA and individual sgRNAs) followed by Sanger sequencing and BEAT analysis after 4 days and flow cytometry analysis after 6 days. **(A)** Sanger sequencing and BEAT analysis of *B2M* on-target editing in T cells. **(B)** Flow cytometry histograms demonstrating *B2M* protein loss. **(C)** Sanger sequencing and BEAT analysis of *REGNASE-1* on-target editing in T cells. N=1.

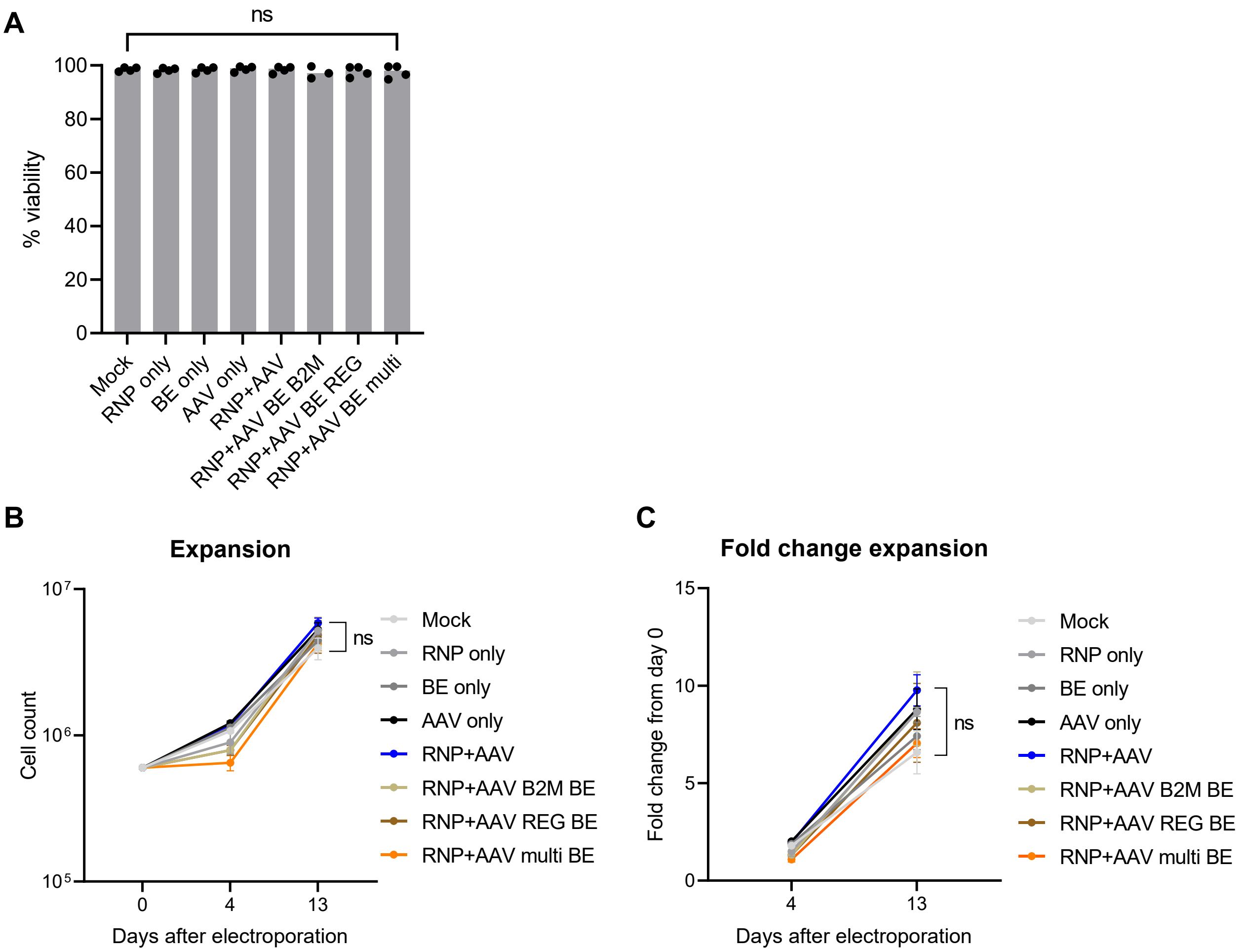

FIGURE S5: Multiplex base editing and targeted integration do not reduce T cell viability or proliferation potential. (A) Viability of primary human T cells assessed 4 days after electroporation. **(B)** Total cell counts assessed by flow cytometry on the indicated days after electroporation. **(C)** Fold change in T cell expansion from day 0 based on cell counts assessed by flow cytometry. Bars represent means. Graphs represent means with SD. N = 4 individual T cell donors. Statistical analysis of base editing data was performed using one-way ANOVA. Statistical analyses of expansion data was performed using two-way ANOVA.

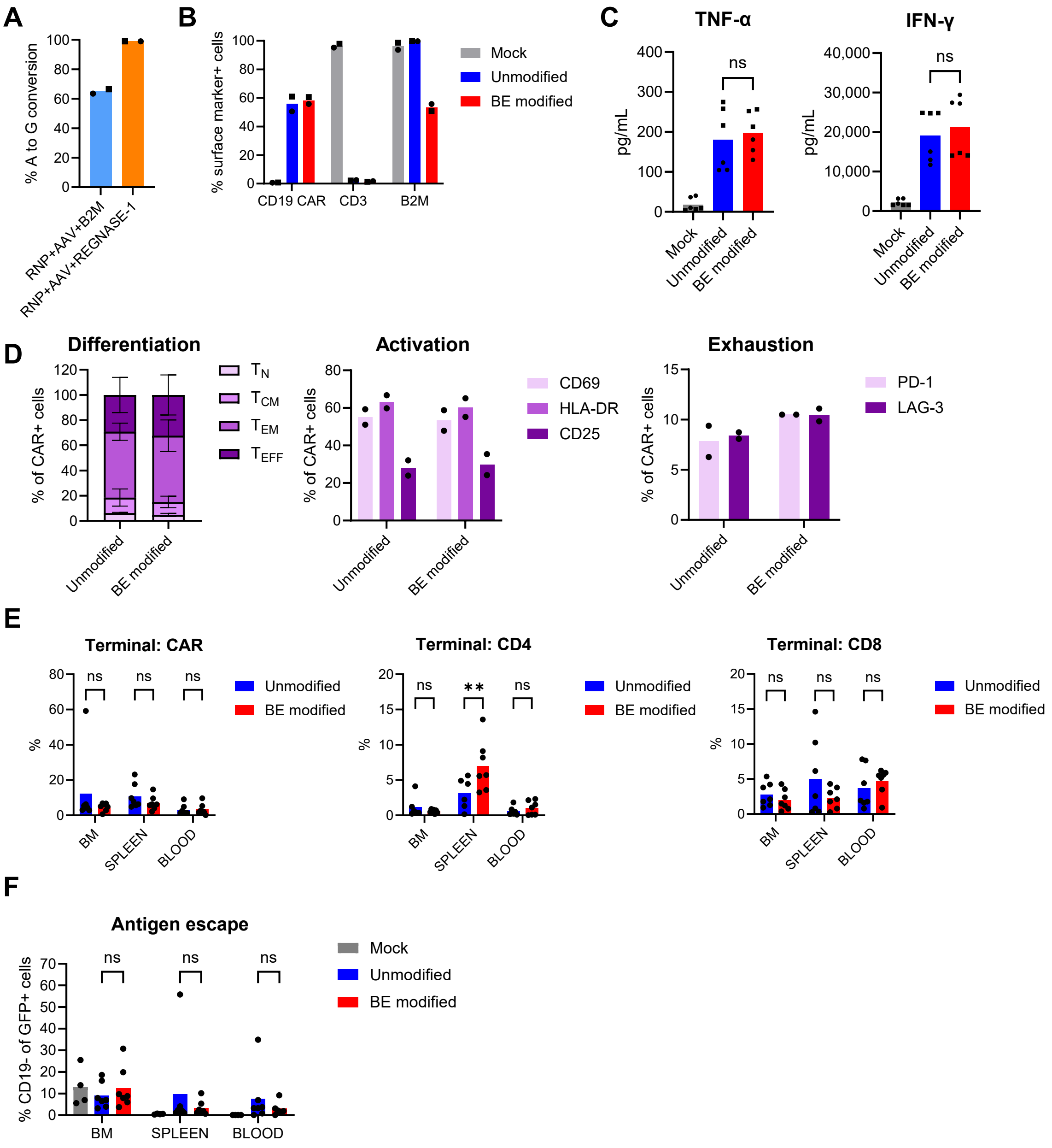

FIGURE S6: Functional assessment of multiplex edited CD19 CAR T cells *in vitro* and *in vivo*. (A) Sanger sequencing BEAT analysis of on-target *B2M* and *REGNASE-1* base editing in primary human T cells. (**B**) Flow cytometry analysis of expression of CD19 CAR, CD3, and B2M for unedited T cells (mock), unmodified CD19 CAR T cells (unmodified), and multiplex base-edited CD19 CAR T cells (BE modified). **(C)** Cytokine secretion (TNF-α and IFN-γ) following cytotoxicity assay measured by ELISA. Supernatants were collected after 24 hours co-culture of unmodified or BE modified CAR T cells with NALM6 CD19+ target cells at the 5:1 effector-to-target (E:T) ratio. **(D)** Phenotypic analysis of differentiation state (CCR7, CD45RA), activation status (CD69, CD25, HLA-DR), and exhaustion markers (PD-1, LAG-3). TN = Naïve T cells (CCR7+CD45RA+), TCM = Central memory T cells (CCR7+CD45RA-), TEM = Effector memory T cells (CCR7-CD45RA-), TEFF = Effector T cells (CCR7-CD45RA+). **(E)** Terminal flow analysis of bone marrow (BM), spleen, and peripheral blood (blood) assessing the frequency of CD19 CAR+ cells, CD4+, and CD8+ T cells out of all live cells. **(F)** The frequency of CD19-negative GFP+ cells was determined to evaluate antigen loss as a potential escape mechanism. Bars represent means. Statistical analyses of cytokine secretion were performed using one-way ANOVA. Statistical analysis of CD19 antigen loss was performed using two-way ANOVA. Significance based on p values was determined as follows: ns, not significant; $*p < 0.05$; $**p < 0.01$; $***p < 0.001$; $****p < 0.0001$. N = 2 individual T cell donors for *in vitro* experiments (N = 3 replicates for 2 individual T cell donors for ELISA data in figure S6C), one donor is marked with circles and the other donor marked with squares. N = 4-7 individual mice for *in vivo* experiments.

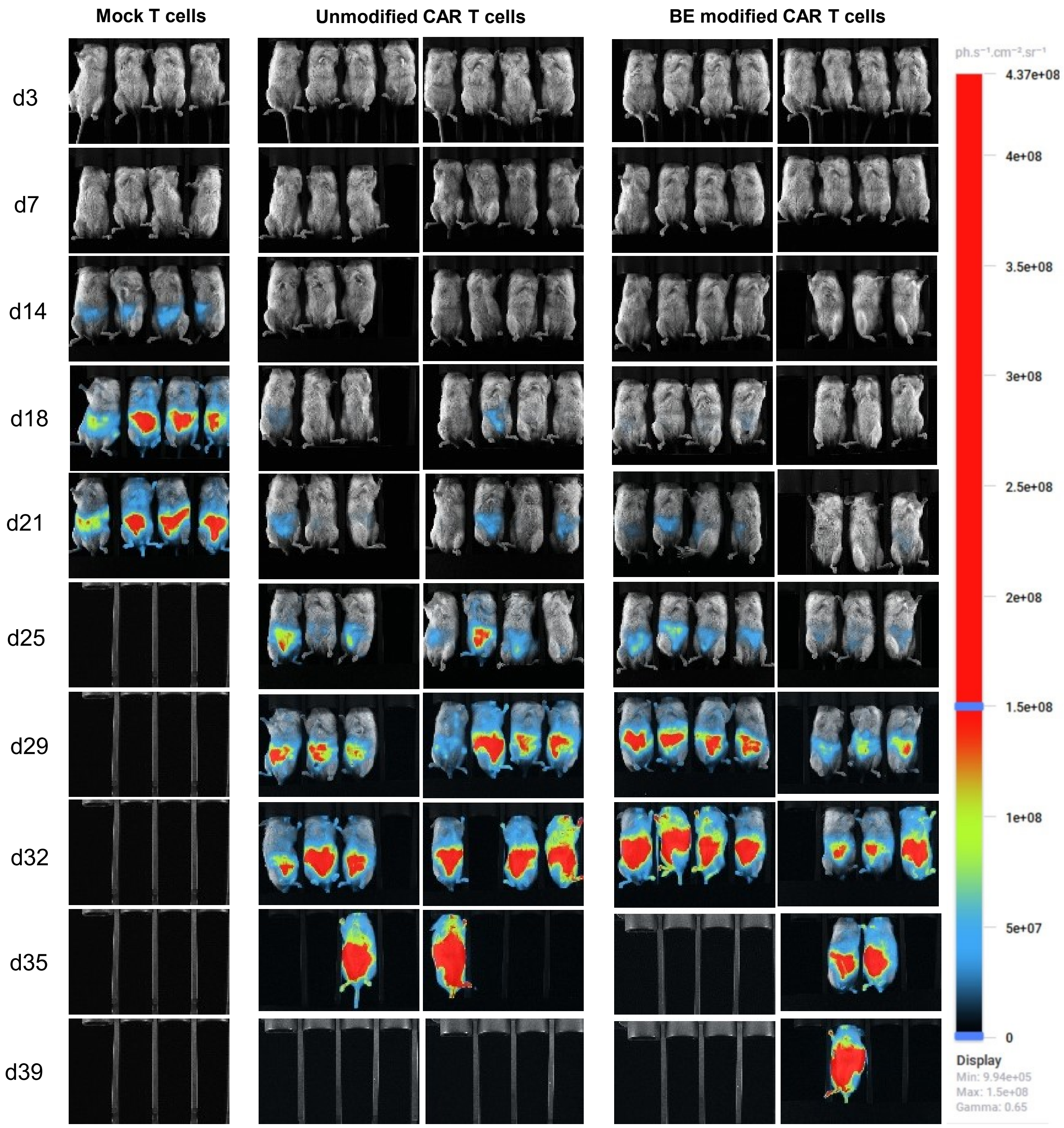

FIGURE S7: All biweekly bioluminescence images of mice related to Figure 3.

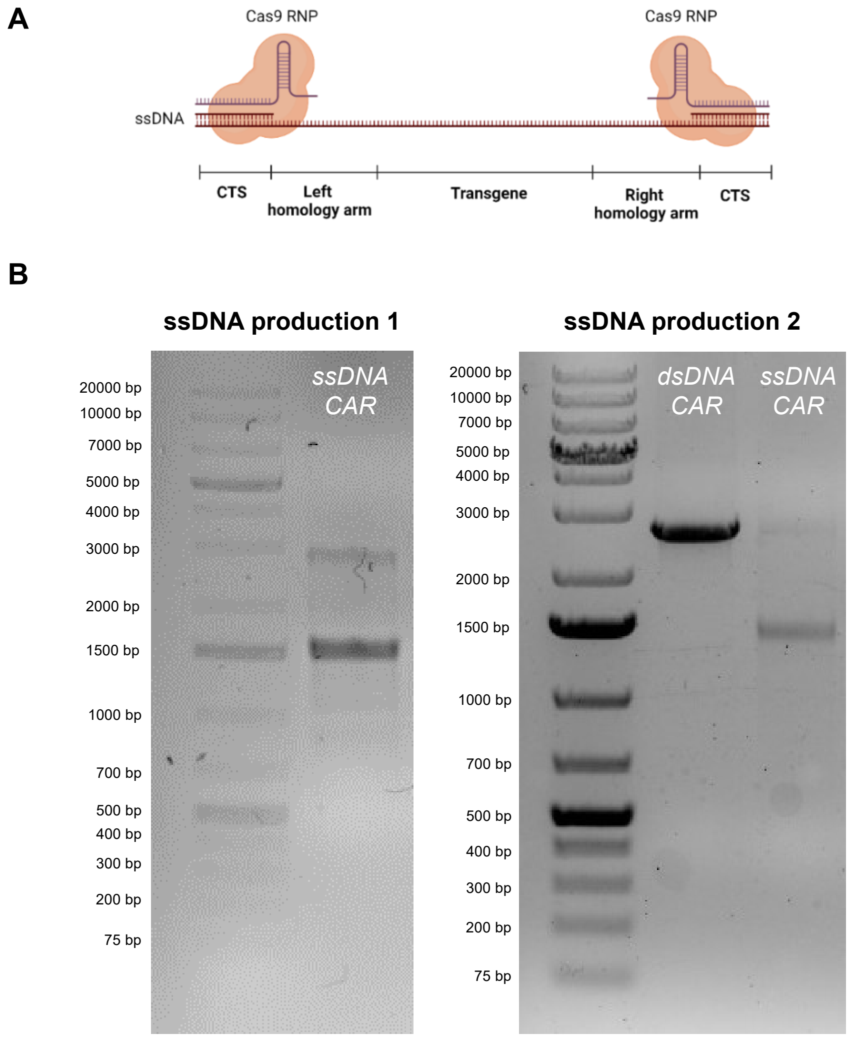

FIGURE S8: Verification of ssDNA integrity and quality. (A) Schematic of ssDNA HDR template with Cas9 target sites (CTS). **(B)** ssDNA productions (located at ~1,500 bp on gel) by comparison with dsDNA (2,660 bp) verified by agarose gel electrophoresis.

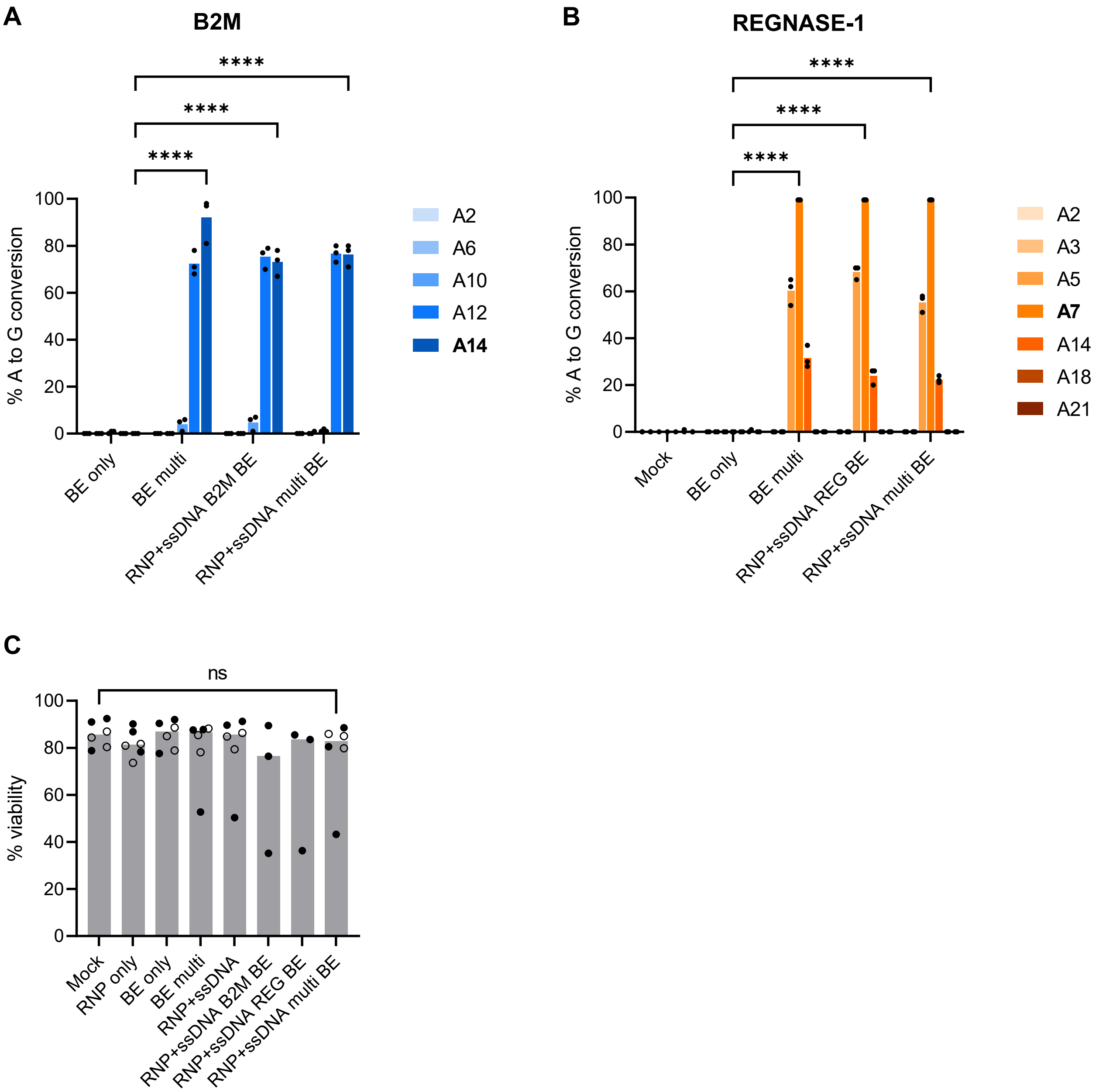

FIGURE S9: Multiplex base editing and targeted integration do not reduce editing frequencies or T cell viability. (A) Sanger sequencing and BEAT analysis of *B2M* editing in T cells. The target adenine (A14) is highlighted in bold and surrounding adenines are included in the analysis. **(B)** Sanger sequencing and BEAT analysis of *REGNASE-1* editing in T cells. The target adenine (A7) is highlighted in bold and surrounding adenines are included in the analysis. **(C)** Viability of primary human T cells assessed four days after electroporation. Bars represent means. Empty circles represent delivery of 1 ug of each base editing reagent and full circles represent delivery of 3 ug of each base editing reagent. Bars represent means. Statistical analyses of base editing data were performed using two-way ANOVA. Statistical analyses of viability were performed using one-way ANOVA. Significance based on p values was determined as follows: ns, not significant; $*p < 0.05$; $**p < 0.01$; $***p < 0.001$; $****p < 0.0001$. N = 3-6 individual T cell donors.

A

Target	sgRNA sequence	PAM	MIT specificity score	Off-targets with 0-1-2-3-4 mismatches	Off-targets next to PAM with 0-1-2-3-4 mismatches
B2M	CACGGAGCGAGACATCTCGGC	CCGAAT	99	0-0-0-0-3	0-0-0-0-0
REGNASE-1	GAACATACTTGTCATTGACGA	AGGAGT	76	0-0-0-1-46	0-0-0-0-0

B

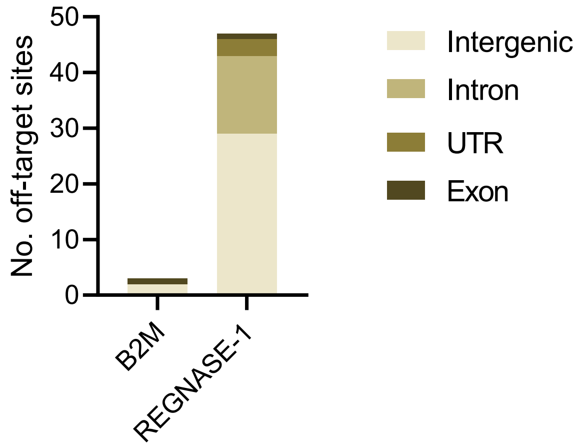

C

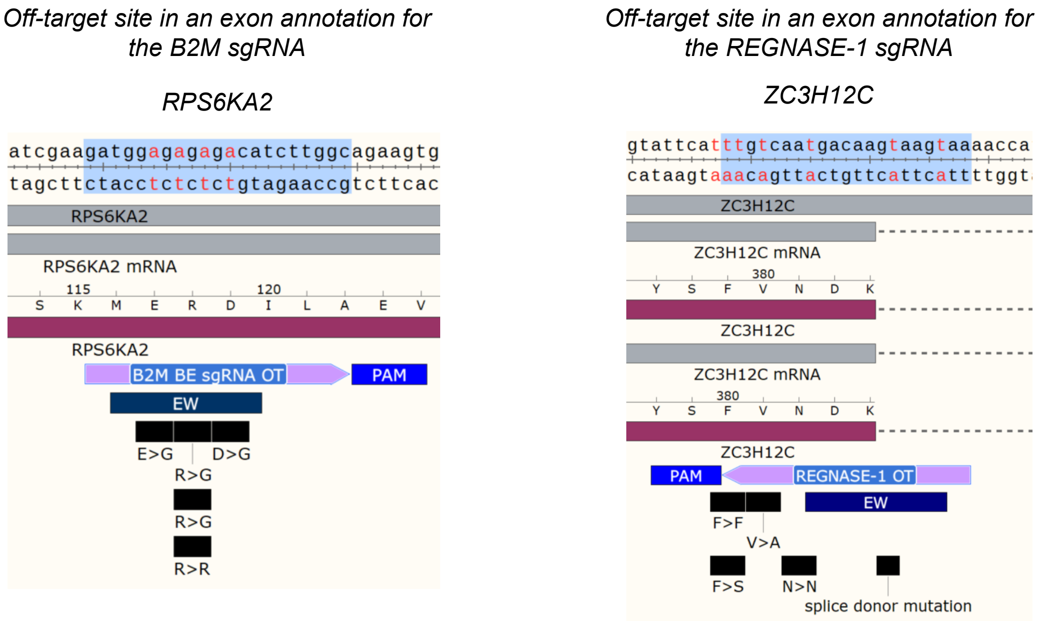

FIGURE S10: Off-target analysis of SaCas9 adenine base editor sgRNAs for *B2M* and *REGNASE-1* knockout. (A) Off-target analysis by the CRISPOR web tool. **(B)** Potential off-target sites plotted based on genomic target location (intergenic, intron, UTR, or exon). Five off-target hits within exons were reclassified as targeting either introns or UTRs upon genomic sequence inspection. **(C)** Off-target site in an exon annotation for the B2M sgRNA (left) and the REGNASE-1 sgRNA (right) and their potential mutational consequences on the amino acid level. OT = off-target, EW = editing window.

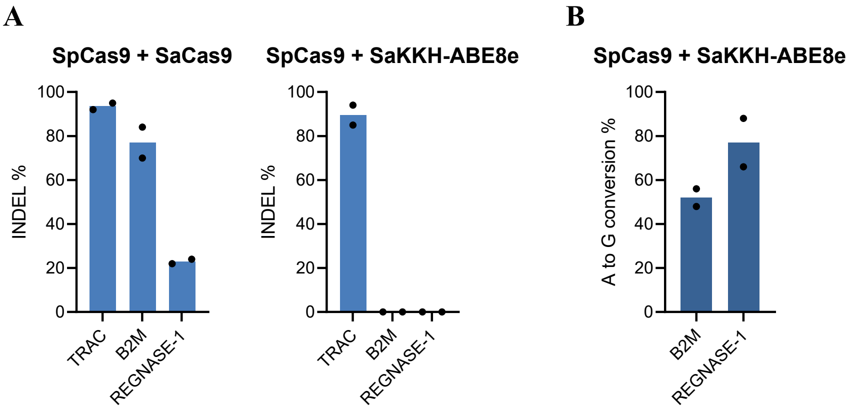

FIGURE S11: INDELs and base editing confirmed in samples prior to ddPCR-based assessment of translocations. (A) Sanger sequencing by ICE analysis of INDELs in T cells electroporated with the SpCas9 + SaCas9 nucleases (left) or the SpCas9 nuclease + SaKKH-ABE8e base editor (right). **(B)** Sanger sequencing and BEAT analysis of base editing frequencies at the *B2M* and *REGNASE-1* loci in T cells electroporated with the SpCas9 nuclease + SaKKH-ABE8e mRNA base editor. Bars represent means. N = 2 individual T cell donors.

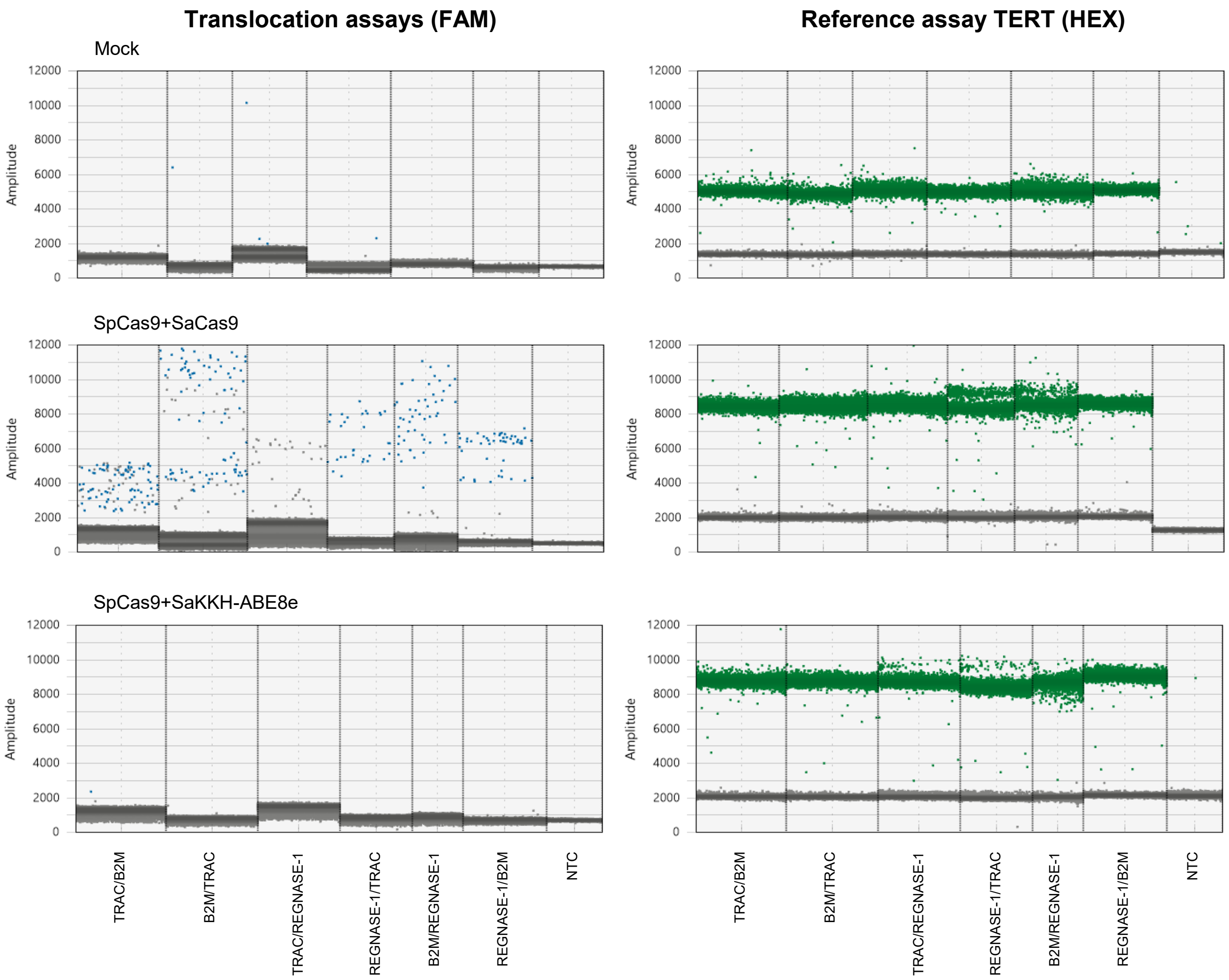

FIGURE S12: Representative ddPCR plots. Representative 1D plots of ddPCR assays to quantify the six balanced translocations for Mock, SpCas9+SaCas9, and SpCas9+SaKKH-ABE8e samples. Translocation assays utilize a FAM probe and the reference assay for the *TERT* locus utilizes a HEX probe. NTC = non template control.

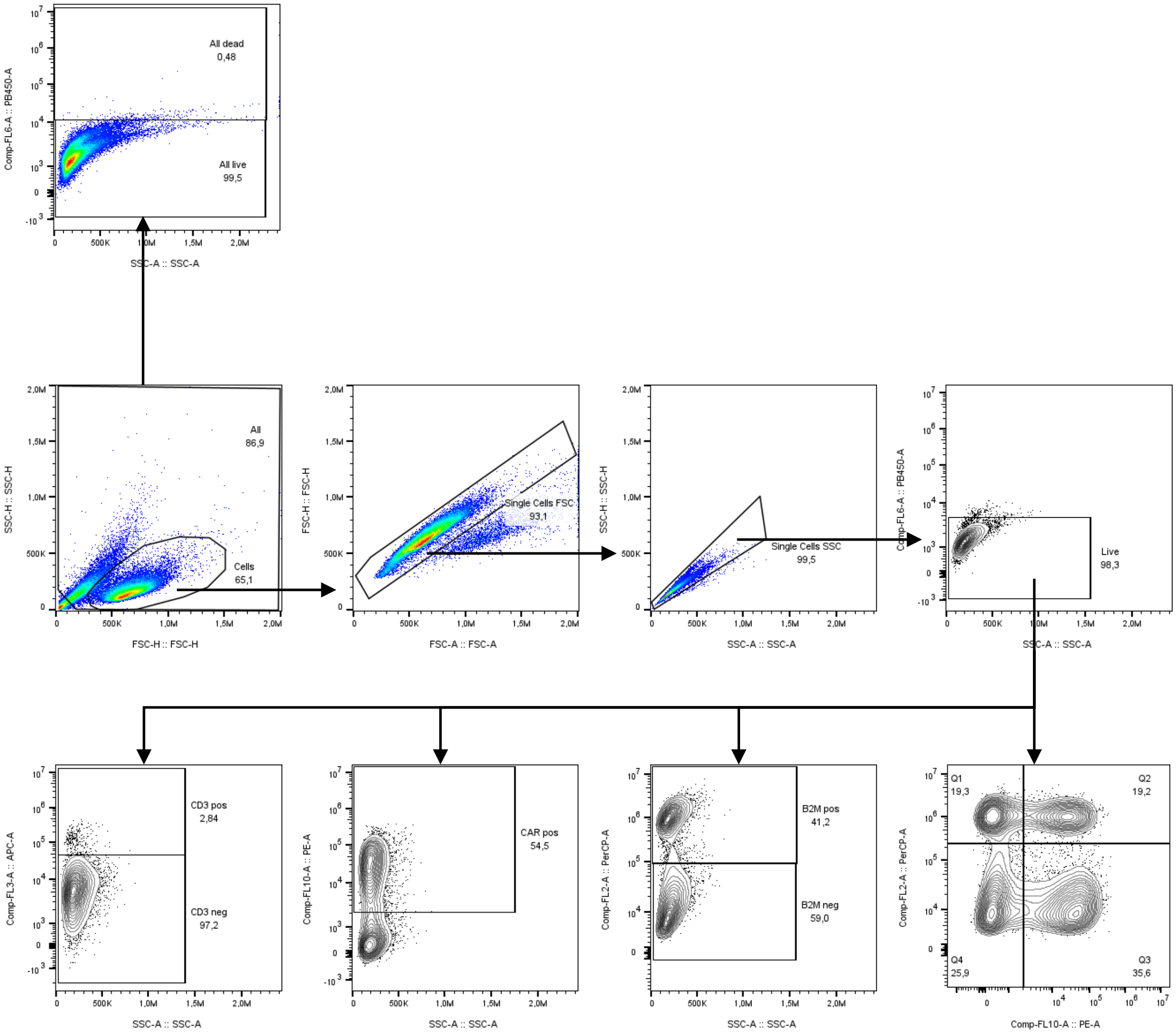

FIGURE S13: Representative flow cytometry gating strategy.

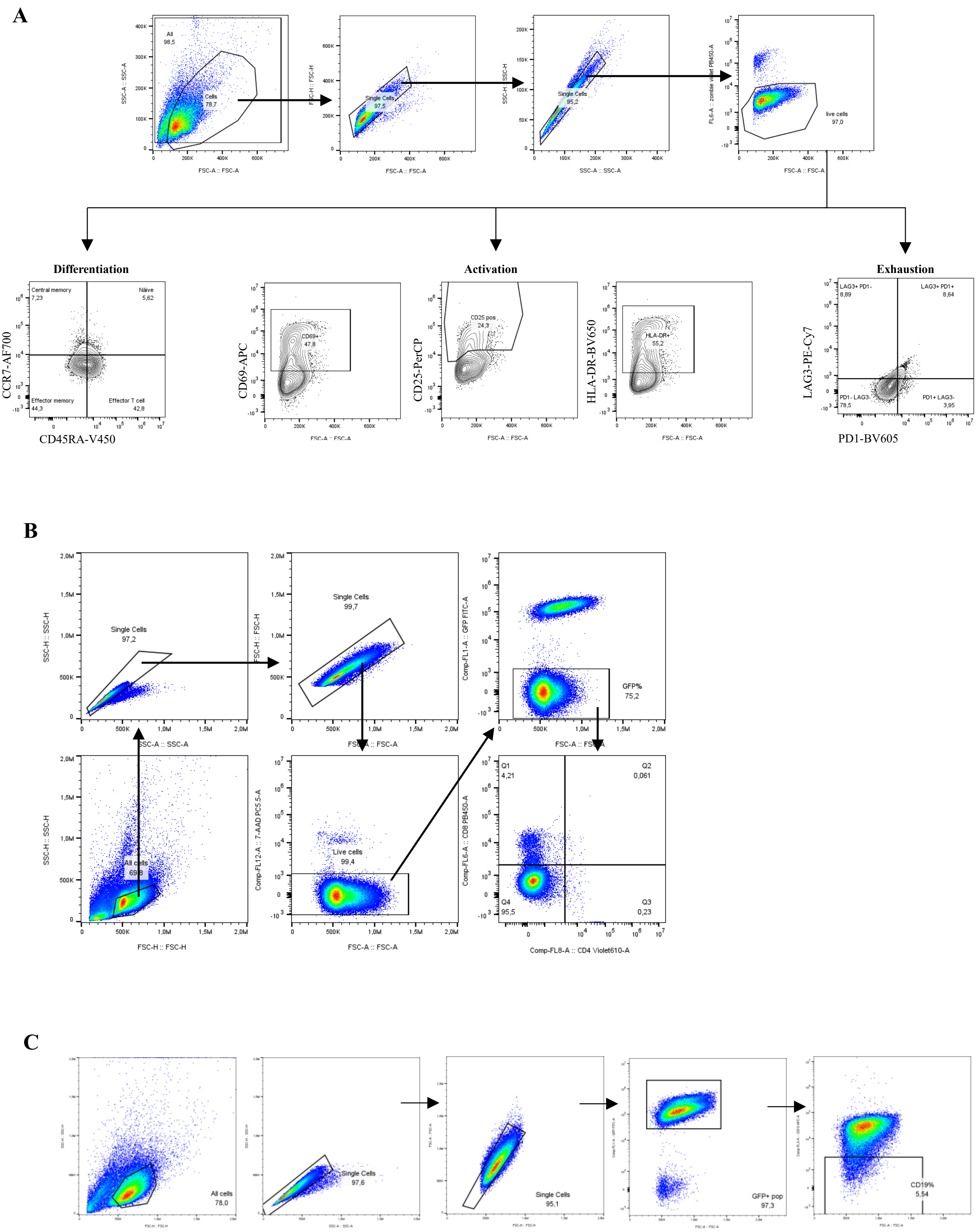

FIGURE S14: Representative flow cytometry gating strategies. (A) Gating strategy for *in vitro* experiments. **(B)** Gating strategy for *in vivo* terminal analysis. **(C)** Gating strategy for *in vivo* antigen escape analysis.

Table S1: sgRNAs for adenine base editing

Screening of adenine base editor sgRNAs (20 nt spacer)					
**Target locus**	**Knockout strategy**	**sgRNA sequence**	**PAM**	**Strand**	**Target base**
B2M	Ex1_START	ACGGAGCGAGACATCTCGGC	CCGAAT	-	13
B2M	Ex2_SA	CGATATTCCTCAGGTACTCC	AAAGAT	+	12
REGNASE-1	Ex3_SA	CCTCCCAGCCATGGGAACAA	GGAGGT	+	7
REGNASE-1	Ex4_SD	AACATACTTGTCATTGACGA	AGGAGT	-	6

Table S2: sgRNAs for adenine base editing with optimal spacer length

SaCas9 adenine base editing sgRNAs (21 nt spacer)					
**Target locus**	**Knockout strategy**	**sgRNA sequence**	**PAM**	**Strand**	**Target base**
B2M	Ex1_START	CACGGAGCGAGACATCTCGGC	CCGAAT	-	14
REGNASE-1	Ex4_SD	GAACATACTTGTCATTGACGA	AGGAGT	-	7
**SpCas9 nuclease sgRNAs (20 nt spacer)**					
**Target locus**	**Target exon**	**sgRNA sequence**	**PAM**	**Strand**	
TRAC	Ex1	GAGAATCAAAATCGGTGAAT	AGG	-	

Table S3: Primers for assessment of editing efficiency

Primers for assessment of INDELs or adenine base editing efficiency			
**Target locus**	**Knockout strategy**	**Forward primer**	**Reverse primer**
TRAC	INDEL	AAGCATGAGACCGTGACTTG	CTGCCAGAACAAGGCTCACT
B2M	Ex1_START	TGAAGTCCTAGAATGAGCGC	TCGACGCCCTAAACTTTGTC
B2M	Ex2_SA	GAAGGTGGAAGCTCATTTGG	GCATCAGTATCTCAGCAGGT
REGNASE-1	Ex3_SA	TCAGGAGATGGAGCCTCAGG	TGACCACCATTCAGAGCAGG
REGNASE-1	Ex4_SD	GCATGGAAGAGGCCTCAGC	GGCCACCTGGAACCATCAAC

Table S4: Primers and oligos for ssDNA production

Primers and oligos for ssDNA production: CD19-CAR		
**Purpose**	**Modification**	**Sequence**
Fw PCR including CTS	5' phosphorylation	CTCTATCAAAATCGGTGAATAGGAGATCCTATTAAATAAAAGAATAAGCAGTA
Rv PCR including CTS	none	CTCTATCAAAATCGGTGAATAGGTGCTTTTTCCCGTGTCATT
5' oligo antisense strand	none	CTCTATCAAAATCGGTGAATAGGAGATCCTATTAAATAAAAGA
3' oligo antisense strand	none	AATGACACGGGAAAAAGCACCTATTCACCGATTTTGATAGAG

Table S5: Primers for Next Generation Sequencing

Primers for NGS		
**Target**	**Type**	**Sequence**
B2M	Forward	GCTGGGCACGCGTTTAATA
	Reverse	TGGAGAAGGGAAGTCACGGA
	Forward + adapter	ACACTCTTTCCCTACACGACGCTCTTCCGATCT GCTGGGCACGCGTTTAATA
	Reverse + adapter	GTGACTGGAGTTCAGACGTGTGCTCTTCCGATCT TGGAGAAGGGAAGTCACGGA
	Forward + adapter + stagger	ACACTCTTTCCCTACACGACGCTCTTCCGATCT C GCTGGGCACGCGTTTAATA
	Reverse + adapter + stagger	GTGACTGGAGTTCAGACGTGTGCTCTTCCGATCT A TGGAGAAGGGAAGTCACGGA
REGNASE-1	Forward	GACACATACCGTGACCTCCAA
	Reverse	CTGGAGCCAAGACCCGCAA
	Forward + adapter	ACACTCTTTCCCTACACGACGCTCTTCCGATCT GACACATACCGTGACCTCCAA
	Reverse + adapter	GTGACTGGAGTTCAGACGTGTGCTCTTCCGATCT CTGGAGCCAAGACCCGCAA
	Forward + adapter + stagger	ACACTCTTTCCCTACACGACGCTCTTCCGATCT C GACACATACCGTGACCTCCAA
	Reverse + adapter + stagger	GTGACTGGAGTTCAGACGTGTGCTCTTCCGATCT G CTGGAGCCAAGACCCGCAA

Table S6: Primers and probes for ddPCR

Primers and probes for ddPCR			
**Target**	**Forward primer**	**Reverse primer**	**Probe**
TRAC	AACCCTGATCCTCTTGTCCC	GCACTGTTGCTCTTGAAGTC	ATCCAGAACCCTGACCCTGCCG
B2M	GTCCCTCTCTCTAACCTGG	TAGGAGAGACTCACGCTGGAT	CCTTGTCCTGATTGGCTGGGC
REGNASE-1	GTTTCCAACGACACATACCG	CGTCTCACTCTCAAAGCTG	CACCTCTGGCTTCCCCTGTGG
TRAC/B2M	AACCCTGATCCTCTTGTCCC	TAGGAGAGACTCACGCTGGAT	ATCCAGAACCCTGACCCTGCCG
B2M/TRAC	GTCCCTCTCTCTAACCTGG	GCACTGTTGCTCTTGAAGTC	CCTTGTCCTGATTGGCTGGGC
TRAC/REGNASE-1	AACCCTGATCCTCTTGTCCC	GTTTCCAACGACACATACCG	ATCCAGAACCCTGACCCTGCCG
REGNASE-1/TRAC	GCACTGTTGCTCTTGAAGTC	CGTCTCACTCTCAAAGCTG	CACCTCTGGCTTCCCCTGTGG
B2M/REGNASE-1	GTCCCTCTCTCTAACCTGG	GTTTCCAACGACACATACCG	CCTTGTCCTGATTGGCTGGGC
REGNASE-1/B2M	TAGGAGAGACTCACGCTGGAT	CGTCTCACTCTCAAAGCTG	CACCTCTGGCTTCCCCTGTGG

Table S7: Antibodies used for flow cytometry

List of antibodies and live/dead markers used for flow cytometry (CytoFLEX S V4-B2-Y4-R3 instrument (Beckman Coulter))					
**Laser nm**	**Antigen**	**Fluorophore**	**Clone**	**Manufacturer**	**Cat#**
405	HLA-DR	BV650	G46-6	BD Sciences	564231
	PD1	BV605	EH12.2H7	Biolegend	329924
	CD4	BV605	OKT4	Biolegend	317438
	CD8	V450	RPA-T8	TONBO Biosciences	75-0088-T100
	CD45RA	V450	H100	TONBO Biosciences	75-0458-T100
	Zombie Violet fixable viability dye	N/A	N/A	Biolegend	423113
	Zombie Yellow fixable viability dye	N/A	N/A	Biolegend	423103
488	LAG3	PerCP-Cy5.5	11C3C65	Biolegend	369311
	B2M	PerCP-Cy5.5	B2M-01	Abcam	ab157311
	CD25	PerCP	M-A251	Biolegend	356132
	CD3E	FITC	OKT3	TONBO Biosciences	35-0037-T100
561	FMC63 (CD19 CAR)	PE	Y45	Fischer Scientific	17101491
	TIM3	PE-Cy7	F38-2E2	Biolegend	345014
	7-AAD fixable viability dye	N/A	N/A	Fisher Scientific	00-6993-50
638	PD1	AF700	Biolegend	Thermo Fisher	329952
	CCR7	AF700	Biolegend	Thermo Fisher	353244
	CD69	APC	FN50	TONBO Biosciences	20-0699-T025
	CD3	APC	UCHT1	TONBO Biosciences	20-0038-T100
	CD19	APC	HIB19	BD Pharmingen	561742
	HLA-A/B/C	APC-Cy7	W6/32	Biolegend	311426
